# Function Follows Form: Using the Aesthetic Association Principle to Enhance Haptic Interface Design

**DOI:** 10.3389/fpsyg.2021.646986

**Published:** 2021-07-05

**Authors:** Stefan Josef Breitschaft, Claus-Christian Carbon

**Affiliations:** ^1^BMW Group, Munich, Germany; ^2^Bamberg Graduate School of Affective and Cognitive Sciences (BaGrACS), Bamberg, Germany; ^3^Department of General Psychology and Methodology, University of Bamberg, Bamberg, Germany; ^4^Forschungsgruppe EPÆG (Ergonomics, Psychological Æsthetics, Gestalt), Bamberg, Germany

**Keywords:** haptic design, user interface, haptic experience, haptic aesthetics, aesthetic association principle, functionality, haptic feedback

## Abstract

Novel tangible user interface technologies facilitate current trends toward seamless user interfaces. They enable the design of yet unseen interfaces and thus the creation of a new kind of haptic language. In order to use the benefits of a touch-and-feel design for a positive user experience, carefully designed haptic feedback plays an important role by providing aesthetically pleasing and sustainable product features. Haptic feedback may exceed mere acquiring of buttons and input-confirmation but enable orientation and even identification of functionality governed by the haptic impression. We employed the aesthetic association principle as a deeply grounded psychological mechanism that assists effective linkage between haptic form factors and associated functional attributes. In order to illustrate this powerful principle, we analyzed the specific associations between certain main haptic surface qualities and associated functional aspects. In a series of three subsequent studies (Pre-Study 1: *perception*, Pre-Study 2: *similarity*, and Main Study: *association*), we explored paradigmatic associations of that kind to develop guidelines which forms are distinct to be used in interfaces. We show how forms are implicitly categorized into functional qualities (on/off, more-less, selection), using a multidimensional scaling procedure and explore explicit form-functionality associations, using a think-aloud method in the context of an automotive interface. For a series of forms, we revealed clear associative relations to specific functions. We will discuss the general value and opportunities of an association-based approach to user experience in order to create intuitive user interfaces. We will also develop ideas for specific areas of applications.

## Introduction—The Physical Empire Fights Back

Technological advancements in the area of tangible user interface technologies and an increasing desire for dynamic and programmable interfaces capable of dealing with an ever-growing number of functionalities led to the ongoing digitalization of user interfaces—among others in the automotive industry. This digitalization of user interfaces is characterized by a fusion of an interactive and decorative surface, leading to the reduction and extinction of dedicated analog interfaces to create seamless and harmonic interfaces. Analog interfaces are increasingly replaced by seamless touch-sensitive areas at the expense of classical passive haptic controls. Yet, the digitalization of user interfaces comes at a price. Featureless interfaces surfaces neglect the subtlety and hands-on qualities of well-designed haptic surfaces. Mainly, we miss the touch-and-feel aspects we need to create a great user experience and to ensure an efficient and low-error use even in eyes-free operation. This becomes imminent in safety-relevant contexts, such as automotive, in which haptic feedback in touchscreen interfaces could massively improve distraction and eyes-free operation. Usability and user experience are limited to mainly visual information. Hence, an innate part of product experience and also brand identity is lost. Not only since several carmakers are focusing in-car interaction on a single touchscreen, but there is also a call for reinforcing the sense of touch in seamless user interfaces by haptic feedback (Stockburger, 2013). Some carmakers are slowly following backward trends at least for some functionalities (Burgess, 2020). The title of a recent Fjord's (a major design consultancy) campaign explains the comeback of the sense of touch in design very well: “Physical Fights Back” (Fjord., 2020).

Increasing fidelity of haptic technologies, expanding mere vibrations to a rendering of complex surface materials (Breitschaft et al., 2020) and also novel technological manufacturing processes (combining different layers of sensing, lighting, and haptics with a single small-package component), enables unprecedented types of interfaces. Haptics will play a key factor in interface design, becoming an essential part of the identity of a brand and a discriminating factor among different competing products as it has already done quite successfully in the past. An example is an iDrive controller of BMW as a dedicated multifunctional input device for in-vehicle infotainment that soon afterward has been adopted by an array of different brands (Bernstein et al., 2008). Since then, the haptic community has been revolving primarily around technical and functional aspects rather than the experience aspect of haptics. Yet resourceful active haptic technology was used to yield a rather simple, analog metaphor—a button click. With novel haptic technologies, haptic experience in interfaces may not only be restricted to confirmation but give orientation toward otherwise featureless interfaces and help acquiring buttons. By triggering functional associations, they may even enable identification *via* haptics (Heijboer et al., 2019a; Breitschaft et al., 2019b). Obviously, this requires a new kind of haptic language for interface design, which integrates seamlessly with the rest of the user interface, thereby creating a whole new level of experience and aesthetics. In his recent paper “Psychology of Design,” Carbon (2019) promotes the importance of a psychological perspective for optimization of current and development of a novel design. Following the framework of Carbon, in this research, we will take a fundamental psychological turn to assist the path of optimizing the quality and intuitiveness of haptics. A major principle within this framework will be the strategic application of the so-called *aesthetic association principle*, which dates back to the experimental founding of psychology in the 1860s and seems to represent the basis of how we perceive and assess design items.

## Need for High-Quality Haptic Feeling

For a very long time, the development of haptic interfaces in the automotive industries was dominated by the necessity of clear, functional, precise, and pleasing user feedback. In the pre-digital era, this was mostly realized *via* mechanical buttons and switches; later, these properties were simulated by electromechanical devices (Lust and Schaare, 2016). Nowadays, in the drive-by-wire times of digitalization, more and more programmable user interfaces are introduced, mostly being without haptic feedback. But even with the advent of devices with programmable active haptic technologies, which offer a great variety of modes and functions, we lack real haptic feedback as the devices do only provide movements in the dimensions of microns, so are quite marginal in everyday usage. The haptics of mechanical devices still seem to be an important benchmark of high-quality user interface. There engineers try to recreate the feeling of such devices in a digital framework. Creating the feeling of handling something manually gives us the opportunity to use deeply rooted and often practiced mechanical routines. This helps us to reduce the cognitive load and to rely on tactile modes only without compromising our cognitive and visual-perceptual capacities (Grane and Bengtsson, 2013; Petermeijer et al., 2015). This is a promising prerequisite for fast, safe, and errorless handling. The joy of handling mechanical devices, furthermore, adds to the user experience by triggering aesthetic aha moments, increased liking, and even fascination in very well-applied cases (Breitschaft et al., 2019a).

Even the finest crafted mechanical interfaces still show an essential problem: They were hard to keep apart. Although excellently engineered buttons feel great, placed side by side, they feel just the same. What we needed and still need is a clear scheme to distinguish them. And, as we are not interested in investing much time and effort to learn the association between specific haptic qualities (e.g., characteristic form) and the function, which is linked with the specific device, we should use already existing associations—associations that are typical, which are deeply rooted in us. In his early writing called “The Aesthetic Association Principle,” Gustav Theodor Fechner, the pioneer of psychophysics and empirical aesthetics, proposed a modern view on perception by emphasizing the power of associations (Ortlieb et al., 2020). The *Aesthetic Association Principle* (AAP) is based on meaning and purpose that is attached to certain objects by experience. As such, perception of an object is not only based on a pure sensory experience, but everything that is cognitively attached to it: “We do not perceive it with the physical eye, but with a mental eye” (Ortlieb et al., 2020). Context and previous experiences become an inherent part of this exact object. As such, Fechner explained the strength of his approach, comparing the aesthetic quality of an orange compared with a visually similar wooden orange ball. If we trigger very common associations, the *aesthetic association principle* offers the ideal playground to develop a general framework of specific haptic qualities, which can be assigned to certain functions.

## The Aesthetic Association Principle in Haptic Interface Design

The *aesthetic association principle* (AAP) has been widely used in several areas of cognitive sciences, mostly in visual perception, although most authors never mentioned Fechner as the originator of these ideas (see Table 1). Most prominently, Palmer and Schloss (2010) provided a powerful explanatory approach for color preferences based on affective responses of participants to color-associated objects. For example, blue has high preference ratings as it is generally associated with positively connoted objects, such as clear skies. Later on, the same group of researchers also found systematic associative links between different styles of music and color (Palmer et al., 2013; Whiteford et al., 2018).

**Table 1:** A brief overview of studies that are compatible with the aesthetic association principle as proposed by Fechner in the year 1866 (Ortlieb et al., 2020).

**Citation**	**Modality**	**Association**	**Method**	**Results**
Palmer and Schloss (2010)	Visual	Color preference—Valence of color related objects	Free association	Color preference (for example blue) can be explained by valence ratings of color-related object associations, such as skies and water
Palmer et al. (2013)	Visual-Audio	Color-Music	Choose 5 most and least consistent color for music pieces	e.g., faster, major-mode music was linked to brighter and more saturated colors, whereas as
Whiteford et al. (2018)	Visual-Audio	Color-Music	Matching 3 best word samples to music	e.g., loud/heavy music relates to darker/more saturated colors
Guest et al. (2011)	Visual-Haptic	Tactile Surface Attributes-Emotional Descriptions	Pairwise Comparison Multidimensional Scaling	e.g., participants reported more comfort with decreasing roughness
Ludwig and Simner (2013)	Visual-Haptic	Color-Tactile stimuli	Assign color best fitting color to tactile stimuli	e.g., softness was associated with pink
Jraissati et al. (2016)	Visual-Haptic	Color-Haptic Adjectives	Two opposing haptic descriptions, Assign/rate fitting haptic descriptions (1–5) to color patch	e.g., hard and heavy objects were associated with black colors
Iosifyan et al. (2017)	Multimodal-Haptic	Movie-Tactile Surfaces	Rate material and movies using semantic differential Choose best-matching sample to movie	e.g., films tackling the notion of beauty were associated with silk
Hasegawa et al. (2018)	Visual-Haptic	Color-Electrostatic Haptic Feedback	Russel's psychological plane Map stimuli on the coordinate system	e.g., low-frequency impulses were associated with colder colors
Götz (2007)	Visual	Surface design features-UI-functionality	Free association	Certain surface features are linked to a specific interaction
Heijboer et al. (2019a)	Haptic	Vibrational Pattern-Semantic Content	Free association	Specific vibrational pattern were linked to alarm, whereas others produced associations like a spring
Mlakar and Haller (2020)	Visual-Haptic	Textile surface Features-UI-functionality	Observation	e.g., protrusion feels like a button

Fechner already stated that the AAP is not restricted to the visual domain. It is a powerful tool for other domains, such as haptic perception as it constitutes great parts of social communication and interaction (Grunwald, 2017). The sense of touch is also called the “intimate sense” and offers an additional layer of emotionality to be taken care of in UI-Design. This goes way beyond a mere psychophysical approach to map subjective perception of haptic impulses to physical parameters. The AAP has already been widely used in a variety of contexts in haptic research, especially focusing on cross-modal associations. Guest et al. (2011) examined associations between tactile sensory descriptions and semantic as well as emotional descriptions to establish an evidence-based tactile lexicon. For example, comfort was reported with decreasing roughness. Generally, there seem to be systematic links between colors and haptic/tactile stimuli and descriptions (Simner and Ludwig, 2012; Ludwig and Simner, 2013; Jraissati et al., 2016). For example, pink was associated with softness; black was associated with hardness. Jraissati et al. (2016) argue that brightness, chroma, and hue have an influence of color-haptic association. For example, colors associated with adjectives like softness and lightness were brighter than those associated with their verbal counterparts. Iosifyan et al. (2017) examined cross-modal associations of haptic materials with complex multimodal stimuli. The participants were asked to match movie snippets with a certain type of haptic material, e.g., sandpaper and silk. In accordance with previously mentioned studies, softer materials, such as silk, were linked to movies, containing notions of beauty, whereas rough materials, like sandpaper, were linked to movies with notions of ugliness. Similar results for color-haptic associations could even be found with electrically induced electrostatic friction impulses (Hasegawa et al., 2018). Rösler et al. (2009) examined the relationship of force-displacement curves in mechanical buttons and their subjective assessment. For example, a longer button stroke induced a heavier button feeling. Regal et al. (2014) leveraged the implicit association between haptic surface properties and user experience evaluations to use haptic materials for UX assessments instead of questionnaires, i.e., a negative experience was represented by rough sandpaper.

There are different types of methods to reveal semantic associations between semantic and perceptual information (see also Table 1). One of the most used methods involves matching tasks. Participants are required to assign or match the most fitting/consistent descriptors for a specific perceptual stimulus, for example, choose the most consistent color when exploring a particular haptic stimulus. Other approaches involve ranking tasks (Iosifyan et al., 2017). Hasegawa et al. (2018) used a mapping task to check for color-tactile associations. Some studies used a free association (think-aloud) task during stimulus exploration. Association patterns are analyzed by establishing a post-study categorization system for the qualitative descriptions (Heijboer et al., 2019a). Another method to reveal underlying semantic qualities is using a multidimensional scaling procedure (MDS). MDS is a statistical method to visualize similarity/dissimilarity data in a multidimensional space. Similarity data can, for example, be gathered, using a similarity estimation method, classification, or a semantic differential method (Okamoto et al., 2013). A well-established psychological procedure, which offers measurement of implicit attitudes, is the implicit association test (IAT) by Greenwald et al. (1998). Subsequent developments include the multidimensional IAT (md-IAT) (Gattol et al., 2011), the go/no-go-association test (GNAT) (Nosek and Banaji, 2001) or the single-category-IAT (SC-IAT) (Karpinski and Steinman, 2006). All these implicit association test variants assume participants react faster when they perceive strongly associated semantic information. Reaction times are used to determine the strength of association.

With the advent of novel interface technologies, the relationship of device-specific technical parameters and affective evaluations are still not fully understood. Yet development is focused on superficial psychophysical relations of parameters rather than holistic aesthetic evaluations. This is hindering the transition from functional to emotional interfaces (Heijboer et al., 2019b). Heijboer et al. (2019a) found that participants mostly describe piezo-actuated haptic feedback, using affective descriptions. They showed that haptic stimuli consisting of different vibration patterns have the potential to convey emotion- and semantically-rich experiences, like “squeezing a spring” and “crunching a snowball.” The notion of using vibration feedback to convey semantic and functional information in user interfaces has already been followed by multiple research laboratories (Brewster and Brown, 2004; MacLean, 2008; MacLean and Hayward, 2008). They propose the use of *tactons* or *haptic icons* (a systematic vibration pattern based on *amplitude, frequency*, and *duration*) to convey abstract and metaphorical messages, such as systems errors. However, *tactons* do not always follow deeply rooted associations but rather arbitrary connections of vibrational patterns and menu actions, which require learning. Yet MacLean and Hayward (2008) propose measures on how to create an easy-to-learn stimulus set.

In interaction design, the term *affordances*—introduced by Norman (2013) and originating from Gibson (1979) *Ecological Approach to Visual Perception*—is used as a main descriptor for the usability of a product. Affordances describe the association of object properties to functional characteristics of a product. Norman (2008) later revised the term “affordance” and proposed to use *signifiers* to refer to interactive clues as it refers to the initially intended meaning of *perceived affordances* of Norman, i.e., the interactivity of a product, depending on context and a perspective of a user. It is the task of the designer to provide interactive clues and which product properties are suited best for certain kinds of interactions. Carefully designed signifying elements are of special importance in cognitive demanding, multitask environments, such as aviation and transportation. For example, in the “Advisory Circular No. 20–175,” the US Federal Aviation Administration proposes to use consistent surface textures, shapes, and other haptic surface features to make interfaces in aviation cockpits detectable, distinguishable, identifiable, and predictable to ensure efficient, errorless and safe interaction (Federal Aviation Administration, 2011). Mlakar and Haller (2020) presented similar design recommendations for textile interfaces, which also consider using unambiguous forms as a clear call for interaction. They propose a preliminary differentiation of forms that indicate an actuation in the normal or tangential direction. Yet they do not provide an in-depth view on form-functionality associations. In this respect, Götz (2007) speaks about “communicative functions” of traditional automotive control elements, which refer to the signifying character of specific design features for the interaction of basic functional principles: on/off, more/less, cursor/selection. In his study, he found reliable associations between different types of functionality and specific interface design features in the context of “traditional” car interiors. For example, a convex surface geometry invited pressing, whereas a cylindrical form with knurling on the side invited to use as a rotary control. Götz argues visual information is a primary source of making interfaces more usable. In an experimental approach focusing on visual stimuli, the participants were not able to physically interact with the virtually modeled stimuli. This approach neglects the influence of visual-haptic information and, hence, the notion of eyes-free operation that focuses on haptic design to alleviate a visual load. Additionally, previous work on signifying interaction features in an automotive context (Götz, 2007; Mueller, 2016), which dates back to the late 2000s, focused on purely mechanical in-vehicle control elements. Interface technologies, design premises, and, most importantly, interaction habits (note the first iPhone was introduced in 2007) changed quite drastically since then, which makes revisiting the signifying character of a potentially novel form language indispensable. The increasing use of interactive surfaces with little to no haptic feedback increases visual distraction by deteriorating orientation in interfaces (Rümelin, 2014; Eren et al., 2015; Beruscha et al., 2017). Passive haptic form features can be a powerful design feature to ease orientation in switch and seamless interfaces. So-called search haptics (Breitschaft et al., 2019a) has primarily been focusing on providing the ability of eyes-free operation by a mere discrimination of buttons, using arbitrary form and edges. By utilizing the AAP to understand the functional character of haptic design features in specialized contexts, such as user interfaces, we suppose that haptic forms can also be used for the identification of basic functional principles. In a preliminary study, Breitschaft et al. (2019b) found differences in the implicit functional associations of different shapes, using a multidimensional scaling procedure.

The goal of this research is to explore signifying haptic forms, more precisely the association of carefully selected forms and their perceived functionality in a user interface context. By applying a multistage experimental study setup (Pre-Study 1: *Perception*, Pre-Study 2: *Similarity* and Main Study: *Association*) we try to (1) to extend the AAP to functional-based associations in haptic design research, (2) give guidelines on discrete, distinguishable, and signifying haptic surface features (see Pre-Study 1), (3) explore the design space of form-functionality associations (see Pre-Study 2 and Main Study), (4) discuss the general value of an association-based approach to user experience design (see section General Discussion), and (5) develop ideas for specific applications on the basis of the concrete associations carved out by our study (see section Ideas for Application).

## Multistage Experimental Setup of The Present Study

**Figure 1:**
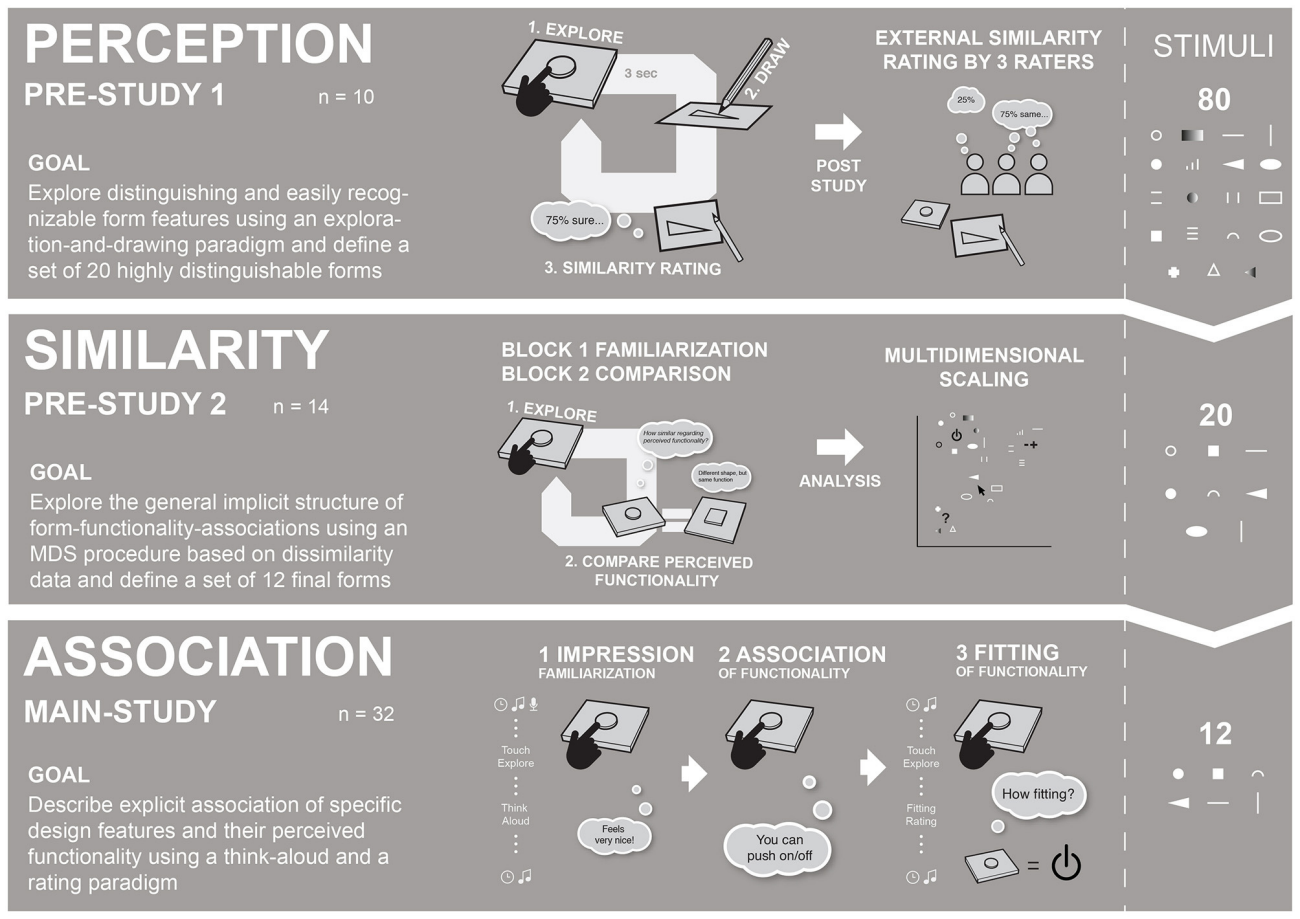
This research utilized a tripartite setup of three subsequent studies: (1) *Perception* included an exploration-drawing paradigm, (2) *Similarity* included a comparison paradigm and (3) *Association* included a think-aloud paradigm.

This paper presents a tripartite multistage approach, consisting of three subsequent studies “*Perception, Similarity*, and *Association*,” as shown in Figure 1. To increase comprehensibility, we will refer to the *Perception* and *Similarity* Studies as Pre-Study 1 and Pre-Study 2 and the Association Study as Main Study.

The initial stimulus set included 80 forms in Pre-Study 1, which was subsequently reduced to 20 forms in Pre-Study and 12 in the Main Study. The restrictions were mainly implemented due to methodological reasons: (1) to ensure an adequate and manageable number of stimuli at every stage and (2) to ensure that, in the Main Study, only a set of highly recognizable and distinguishable forms is used. This research was not intended to provide a fully systematic examination of form-functionality descriptions but to provide an initial implementation of the AAP in a haptic design context.

Pre-Study 1 (see section Pre-Study 1—Perception), *Perception*-Study, was carried out to examine which forms are easy to explore, recognize, and discriminate, using an exploration-and-drawing task. As a result, 20 recognizable and distinguishable forms were identified to be used as stimuli for Pre-Study 2.

Pre-Study 2 (see section Pre-Study 2—Similarity), *Similarity*-Study, utilized a multidimensional scaling (MDS) procedure to explore a general underlying pattern of form-functionality (dis-)associations between different forms. Do they differ at all based on their perceived functionality? A similar approach to “perceptually optimize” a set of stimuli has been reported by MacLean and Hayward (2008). As a result, 12 forms were identified to be used in the Main Study.

Even though having a preliminary character, Pre-Study 1 and Pre-Study 2 yield important insights and prerequisites for the examination of form-functionality associations, which is why they play an inherent part within our highly systematic research approach. In addition, to make the complete stimulus selection process more transparent, the Pre-Studies will be described in more detail. Insights from Pre-Studies 1 and 2 were used to refine and reduce the initial set of 80 forms to 12 highly distinguishable forms that were used in the Main Study.

The Main Study (see section Main Study—Association and Fitting), *Association-*Study, uses a final set of 12 forms and examines explicit form-functionality associations. In the first part of the Main Study, the participants freely reported functional associations, using a think-aloud method. In the second part, they rated the fitting of the forms for functionality categories defined in Pre-Study 2.

In the following section, all the Pre-Studies are described with respect to the implemented method, results, and stimulus selection. The Main Study includes a description of the method, results, as well as a discussion of specific form-functionality associations. The general experimental premises were the same for all three studies: (1) Haptic stimuli were concealed within a touchbox while haptic exploration, (2) the same room, apparatus, and haptic stimuli and general procedure (washing hands, consent form, post-session feedback, etc.) were used during all three studies and (3) all the studies included an introduction focusing on an automotive interaction context to provide a concrete scenario, following the idea of *scenario-based touching* (Jakesch et al., 2011).

## Pre-Study 1—Perception

Haptic forms in user interfaces should be detectable effortlessly within short haptic glances (Klatzky and Lederman, 1995). Moreover, forms need to be distinguishable to retain their unique character (Federal Aviation Administration, 2011; Mlakar and Haller, 2020). In Pre-Study 1, we primarily used qualitative as well as quantitative data to explore design guidelines for the design of haptic forms and to select stimulus material for Pre-Study 2.

### Method

#### Participants

Ten right-handed participants took part in Pre-Study 1. They were between 19 and 36 years old (*M*_age_ = 27.1 years, *SD* = 4.4); six participants were female. All the participants worked in the automotive sector; five had prior experience with haptic feedback. The participants were naïve to the aims of the study and had not gone through special training in haptic perception or drawing. All the participants were right-handed.

#### Apparatus

**Figure 2:**
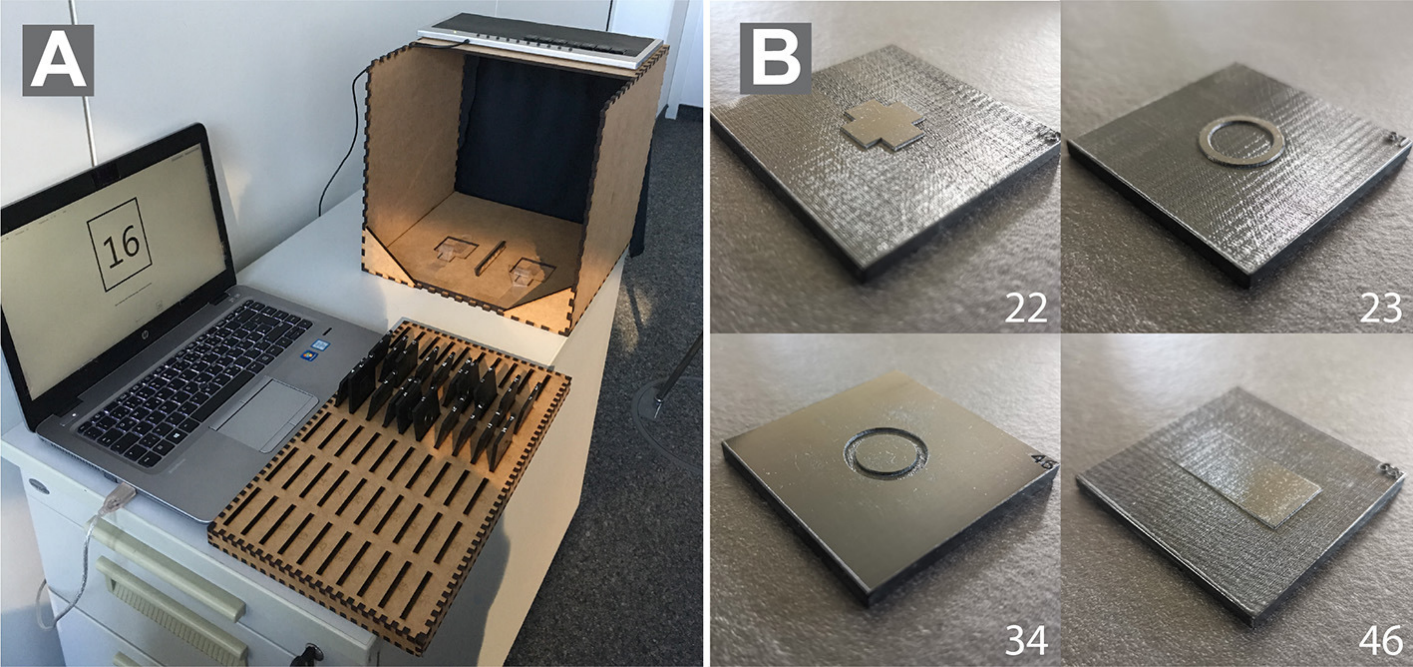
**(A)** The figure shows the view of the experimenters. Haptic stimuli were presented in a custom-made touchbox with a curtain on the side of the participants to ensure pure haptic exploration. **(B)** shows an exemplary stimulus used in the studies. Numbers correspond to the forms from Pre-Study 1.

Stimuli were presented in a touchbox with a curtain in front and an opening in the back (see Figure 2A). The touchbox was positioned like a center console of a car and adjusted to the handedness of the participants. A black curtain prevented the participants to see the stimulus material and enabled them to focus on their haptic impression (see Figure 2). The opening in the back of the box enabled the experimenter to easily exchange stimuli. Different inlays were used over the course of the three studies to accommodate a varying number of stimuli. A little recess in front of every form position on the inlay helped orientation and was used as a common starting point for the exploration phase in all experiments.

#### Stimuli

**Figure 3:**
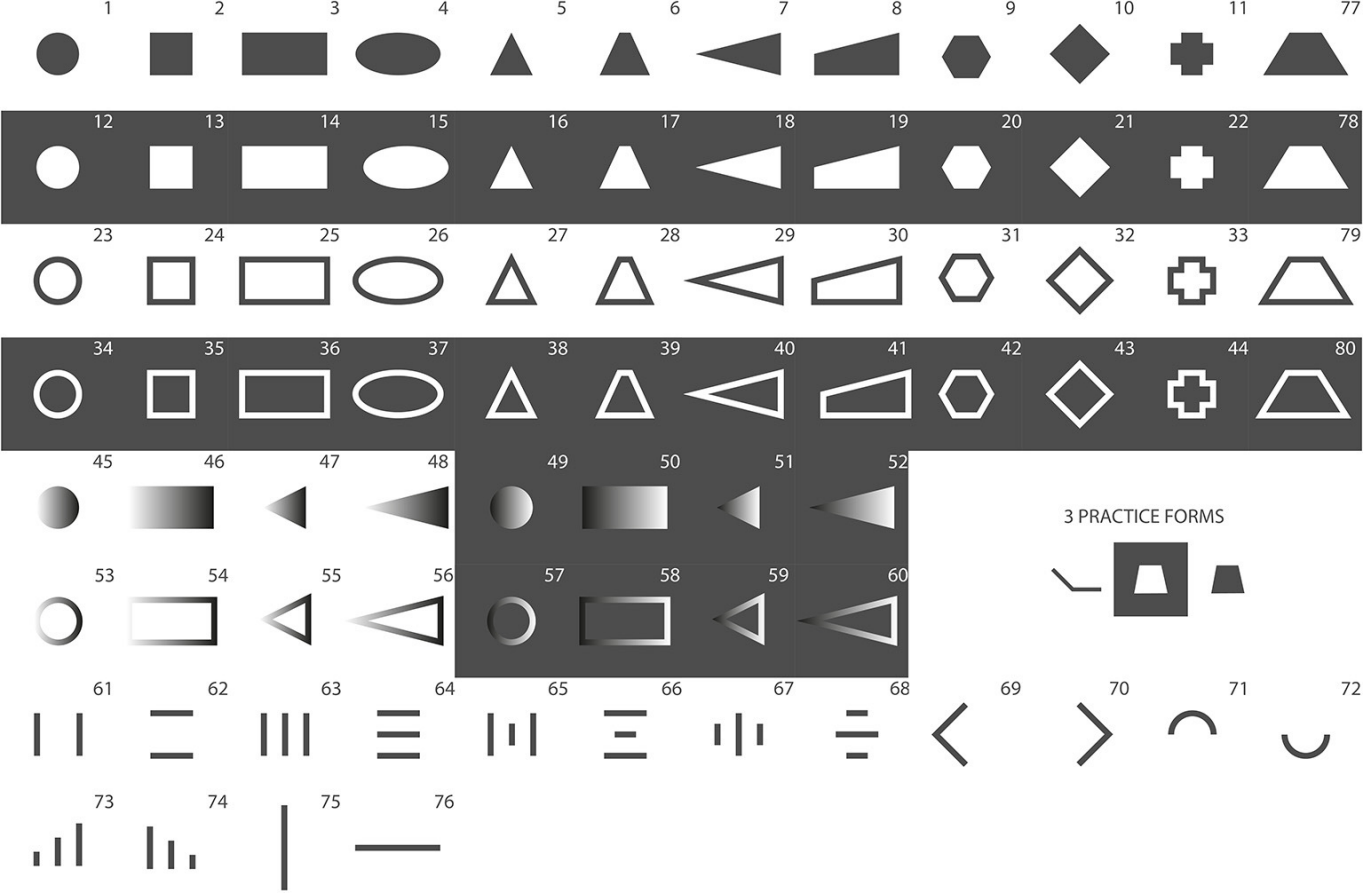
These stimuli present the initial set of 80 stimuli, which was used in Pre-Study 1. Gray areas equal protruded (non-engraved), and white equals laser-engraved areas.

In Pre-Study 1, an initial set of 80 a priori-designed stimuli was used (Figure 3). This study employed a within-subject design, meaning all the participants explored all 80 shapes. The haptic forms were created during a workshop with experts from different professions, including psychology, design, usability, and engineering to create a wide variety of potentially suitable and appealing forms. The set of 80 forms was not based on a fully exhaustive and systematic variation of physical parameters as this was not the goal of this study. Object properties, such as height in the z-axis, size, and surface geometry were deliberately excluded during the process of stimulus creation as it would have increased stimulus complexity. As the stimulus set was refined over the course of this research, numbers of forms indicated in all figures change with every experiment.

The experimental stimuli were made of 50 × 50-mm polycarbonate (pc) plates. The forms were maximum 15 × 15 mm (e.g., Forms 1 and 2), 30 × 15 mm (e.g., Forms 3 and 77–80) or 15 × 30 mm (e.g., Forms 69 and 75) in size. The authors are aware that recognition of forms may depend on the size of haptic forms. The size was chosen based on a compromise of design and usability aspects. A laser engraver was used to manufacture the physical forms, which were previously created in Adobe Illustrator (files are available upon request). The thickness of the pc-plates is 4.11 mm. Engraved areas (depicted in white in all figures) of the pc-plates were 3.66 mm thick, which results in a height of 0.45 mm for the protruded areas (depicted in gray in all figures). Using a laser engraver made the production of forms easy and efficient. Furthermore, polycarbonate is very resistant to abrasion. Engraving created a slight texture on the top surface of the pc-plates. A subtle texture was applied to the protruded areas of the pc-plates in order to reduce friction of the pc surface and create a homogenous feeling while exploring the surface. Figure 2B depicts four exemplary forms that were used in the study.

#### Procedure

**Figure 4:**
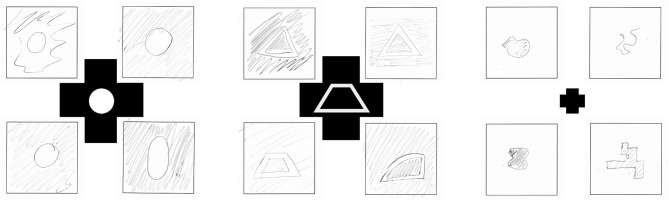
Examples of participant's form drawings in Pre-Study 1. The middle form depicts which form the participants explored.

Pre-Studies 1 and 2 and the Main Study were conducted in the same silent and separated room. Before every experimental session, the participants washed their hands for hygienic reasons and gave consent for taking part in the study before data collection. The participants were seated next to the touchbox, which was placed on a storage container to provide a comfortable height for the participants to explore the stimuli and adjusted to the handedness of the participants. The participants were told to find a comfortable position to reach the stimuli in the touchbox and comfortably switch to the drawing after haptic exploration. A folder containing empty pages to collect the drawings of the participants was placed on a table to the left side of the participants. The participants were introduced to the background and procedure of this pre-study and were instructed how to draw the explored three-dimensional forms. They were told to focus on two-dimensional geometry of the forms in their drawings and to use pencil shading to indicate protruded or recessed areas of the stimuli. Darker shading indicated recessed areas, whereas lighter shading and blank spaces indicated protruded areas of the form (see Figure 4). A short training session, including three forms, helped the participants to practice the drawing procedure. A single trial included a tripartite task: (1) haptic exploration in 3 s, (2) drawing the explored shaped, and (3) a subjective rating (see Figure 1). The experimenter started the trial once the participants placed their fingers on the starting point in the touchbox. The first acoustic signal marked the beginning of the trial. The participants explored the respective form for 3 s with their index fingers. A second acoustic signal marked the end of the 3-s-exploration phase. Afterward, the participants drew the explored form. Following the drawing, the participants were asked to judge the perceived similarity of the drawn and explored form on a scale from 1, “very different,” to 7, “very similar,” using the following question: “How similar is the form you drew compared with the one you explored?.” In total, every participant completed 80 experimental trials (80 different forms). All haptic forms were presented in a randomized order for every participant. A complete session took ~1 h.

### Results and Stimulus Selection

**Figure 5:**
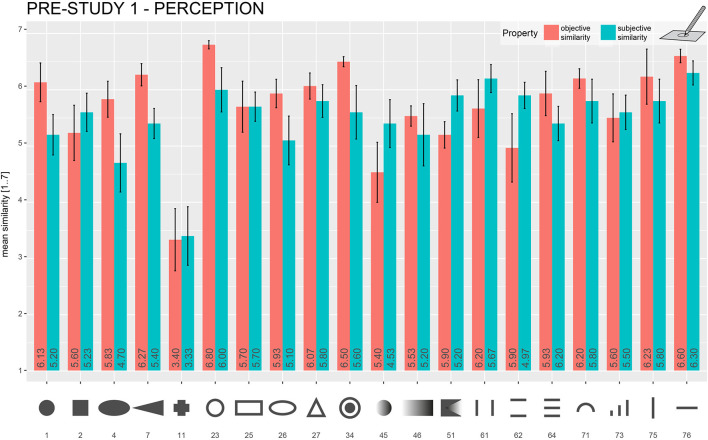
Mean objective and subjective similarity ratings for the explored forms in Pre-Study 1 (*Drawing*) that were chosen to be used in further studies. The mean objective and subjective similarity rating is represented in each bar. Error bars = ±1 standard error of the mean.

Ahead of data analysis, drawings, as well as ratings, were preprocessed, which included scanning and cropping the images. To gain objective similarity ratings, three independent raters (who did not take part in the study) were given all 800 pairs of the drawings and engraving templates of the forms in a post-experimental rating. The task of the rater was to judge the similarity of the two adjacent forms on a seven-point scale with 1 being “very different” and 7 being “very similar,” following this question: “How similar are both forms?.” Mean values for the participants' (subjective) and post-experimental (objective) similarity ratings were calculated (see Figure 5). Due to the limited space, only those parts of the data that were relevant to the stimulus selection were considered and subsequently described on a qualitative level.

In general, the participants performed well in this exploration-and-drawing task. Despite a very short 3-s exploration time, most of the form drawings showed a strong resemblance to the presented stimuli. A general finding was that the quality of form drawing deteriorated with more complex forms. Comparably easy forms, such as a horizontal line (the mean of subjective rating, 6.30/the mean of objective rating, 6.60) and a raised-line circle (6.00/6.80), scored much higher similarity ratings as more complex forms, such as the fully protruded cross (3.4/3.33). A comparison of subjective and objective similarity ratings for the forms that were chosen to be used in Pre-Study 2 is given in Figure 5. Orientation and aspect ratios of forms seem to get lost by drawing. For example, the diamond-shaped stimuli were often recognized as simple squares. Rectangular forms were reproduced with distorted aspect ratios in a lot of cases—mostly as squares—making them easier to confuse with squared forms. Raised-line figures that consisted of a multitude of differently sized lines were also not reproduced correctly in many cases (#61–#74 in Figure 3). Another common observation was that forms with dull angles, such as the upper parts of trapezes, were drawn as semicircles, replacing edges by rounding.

**Figure 6:**
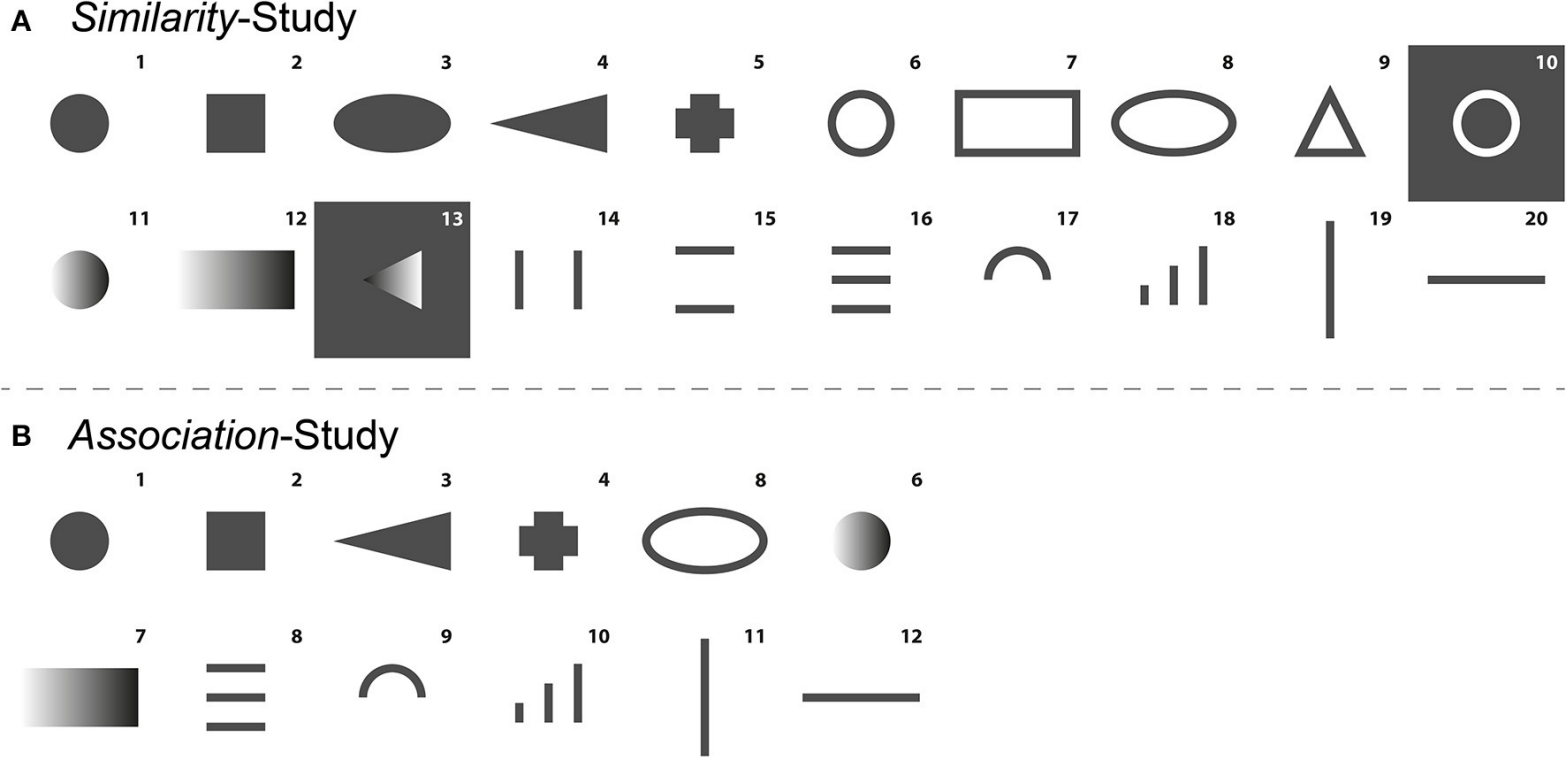
**(A)** Forms (*k* = 20) that were selected to be used in Pre-Study 2. **(B)** Forms (k = 12) that were selected to be used in the *Association*-Study. Gray areas equal protruded (non-engraved), and white equals laser-engraved areas.

Forms were selected based on criteria, such as (1) subjective and objective similarity ratings, (2) distinctiveness from other forms (e.g., rectangular and squared forms were often confused) and (3) advice from tangible user interface experts regarding suitability and feasibility in user interfaces. Figure 6A shows the 20 forms that were selected to be used in Pre-Study 2.

## Pre-Study 2—Similarity

Pre-Study 2 focused on examining general underlying form-functionality associations of 20 previously described haptic forms, using a multidimensional scaling procedure (MDS). Similarity data were collected, using a form-comparison paradigm. We also used Pre-Study 2 to select a set of 12 final stimuli for the Main Study.

### Method

#### Participants

Fourteen right-handed participants took part in Pre-Study 2. They were between 21 and 57 years old (*M*_age_ = 29.9 years, *SD* = 9.1). Six participants were female. All the participants worked in the automotive sector. The participants were naïve to the goals of the study. Three participants already took part in Pre-Study 1. Four participants had prior experience with haptic technology. All the participants were right-handed.

#### Apparatus and Stimulus

Pre-Study 2 utilized the same apparatus, general setting experimental material as in Pre-Study 1. The inlay of the touchbox was adapted to fit two adjacent forms. The forms were separated by a small detent. The touchbox was adapted to the dominant hand of the participants. The MDS procedure requires similarity data from all form pair combinations. The stimulus set derived from Pre-Study 1 consisted of 20 forms (see Figure 6), resulting in 190 (undirected) pair combinations and comparisons.

#### Procedure

The participants were introduced to the background and procedure of Pre-Study 2. They were asked to imagine encountering these shapes in a future user interface. The experiment included a familiarization and comparison phase. In the familiarization phase, the participants were able to familiarize themselves with the stimulus set. They were instructed to freely explore all the forms one by one, using their index fingers and think of which kind of functionality the forms could represent in a user interface without speaking out loud. In the comparison phase, two adjacent forms were presented simultaneously. All haptic forms were presented in a randomized order for each block. The participants were asked to freely explore both forms. There was no time restriction for the exploration phase. The participants were asked to answer the following question after exploration: “How similar are both forms in terms of their perceived functionality on a scale from 1 (not similar at all) to 7 (very similar)?” After completion of all the comparisons, the participants were given a short questionnaire, containing illustrations of all the forms. The participants were asked to write down their initial form-functionality associations. These insights were gathered to have an initial starting point for the interpretation of the multidimensional scaling procedure. A complete session took ~75 min.

### Data Analysis and Results

**Figure 7:**
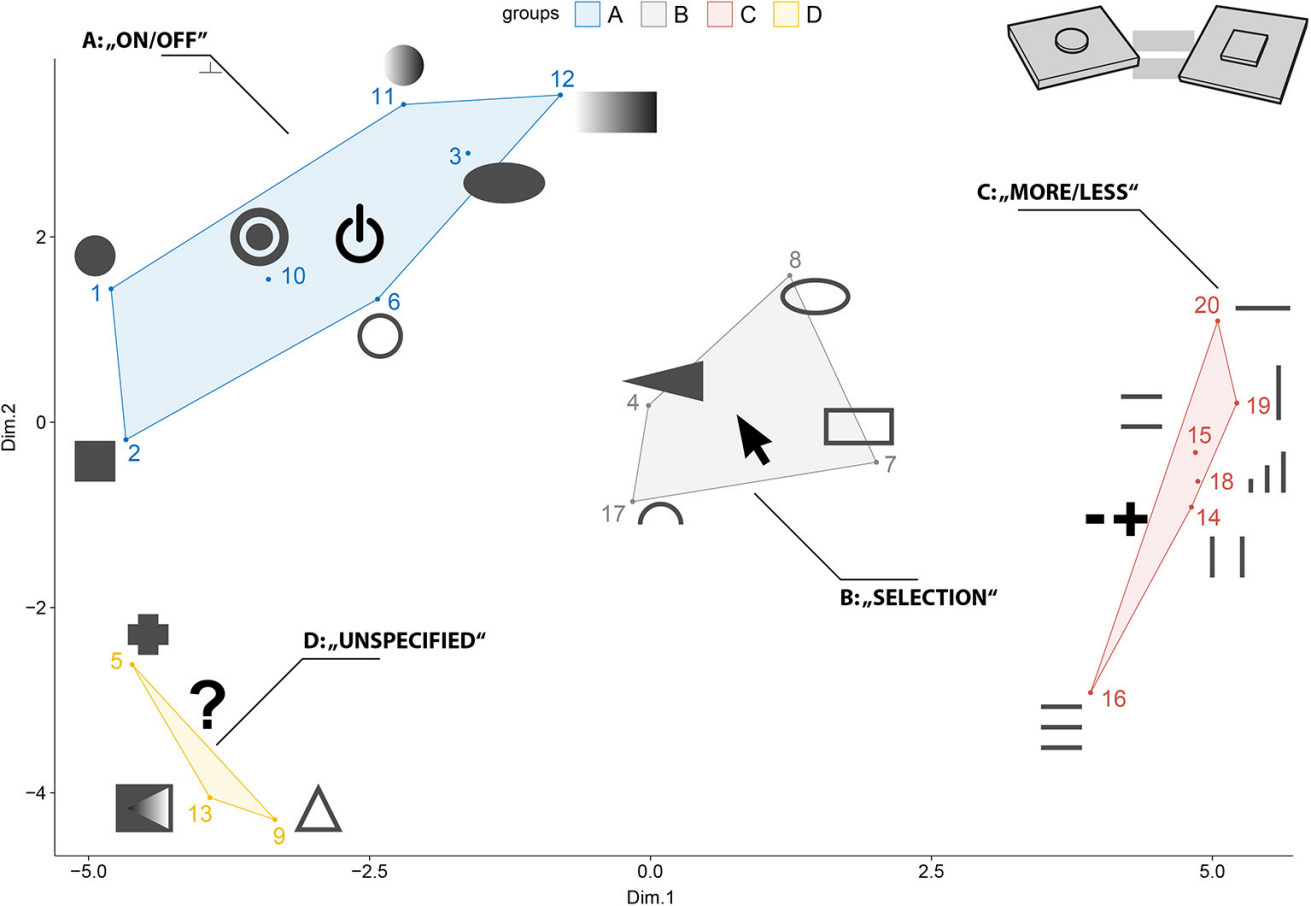
The MDS procedure yielded four clusters that were interpreted with the following functional categories: **(A)** “on/off,” **(B)** “selection,” **(C)** “more/less,” **(D)** “unspecified”.

The multidimensional scaling procedure (MDS) allows to explore implicit similarity structures of different objects and visualize their reciprocal relationship based on pairwise (dis-)similarity measures (Jaworska and Chupetlovska-Anastasova, 2009). The MDS has previously been used to understand basic principles in haptic perception (Cooke et al., 2010; Okamoto et al., 2013). In the context of this study, the MDS was used to explore the general, underlying association pattern of forms and their perceived functionality. For data preparation, ratings of the pairwise comparisons were averaged across all the participants. Afterward, the data were transformed into a dissimilarity matrix, representing empirical distances between each of the form combinations. The MDS was performed on the dissimilarity matrix, using the *cmdscale()-*function in the statistical software R version 4.0.2 (R Core Team., 2021). We also used the *goeveg* (Friedemann and Schellenberg, 2021) and *igraph*-package (Csardi, 2019). An a priori stress test was performed to determine the number of clusters that needed to be extracted. The stress test (Kruskal's *Stress-1* < 0.2) revealed four clusters to be a reasonable number of clusters to represent the original space of stimuli. Figure 7 depicts the configuration gained from the MDS. For ease of interpretation, the forms were directly embedded in the visual presentation of the MDS configuration. Using the initial impressions of the participants reported in the post-experimental questionnaire, and also results from Götz (2007) and Mlakar and Haller (2020), we interpreted the four clusters, using the following categories: (A) “on/off,” (B) “selection,” (C) “more/less,” (D) “unspecified” (see Table 2).

**Table 2:** Preliminary interpretation of the MDS results as given in Figure 7.

**Cluster**	**Description**	**Forms**
A	**On/Off** regarding confirmation by pushing	**1**, **2**, 3, 6, 10, **11**, **12**
B	**Selection** regarding selecting items from a horizontal or vertical list by pushing or sliding	**4**, 7, **8**, **17**
C	**More-or-Less** regarding sliding and rocker type switches	14, 15, **16**, **18**, **19**, **20**
D	**Unspecified** with ambiguous associations	**5**, 9, 13

*Form numbers in bold indicate forms that were chosen to be further used in the Main Study*.

### Discussion and Stimulus Selection

Pre-Study 2 aimed to investigate the general implicit structure of form-functionality associations. Judging from the MDS plot (see Figure 7), there seem to exist four distinguishable clusters with respect to form-functionality associations: (A) “on/off,” (B) “selection,” (C) “more/less,” and (D) “unspecified.”

Different forms within a cluster share a common functional association, for example, different raised-line patterns—regardless of orientation—seem to invite sliding. It also seems reasonable to assume that there is an underlying pattern of interaction within the MDS configuration. Dimension 1 might correspond to spaceness of form and Dimension 2 to the roundness of form. Forms that were mostly related to sliding can be found on the positive side of the x-axis, whereas forms on the negative side of the x-axis were mostly related to pushing in the initial impressions of the participants. Forms within the “unspecified” cluster either do not activate specific functionality associations due to the fact of being too complex or contain a multitude of signifying design features, which makes them ambiguous with respect to functionality and interaction.

Only a selection of forms from every functional cluster was used in the Main Study. Forms were chosen based on (1) their location in the MDS configuration, (2) their distinguishing character and (3) feasibility in a user interface. In addition, forms 10 and 13 were the only forms, including recesses, and thus were excluded from the selection to avoid any biases in the final *Association*-Study. Based on the MDS configuration, the following forms were selected: 1, 2, 4, 5, 8, 11, 12, 16, 17, 18, 19, and 20 (see Table 2).

## Main Study—Association and Fitting

The MDS procedure in the previous *Similarity*-Study only depicts the configuration of the underlying similarity structure of the haptic forms. It is a mere description and does not provide any information as to which forms and design features correspond to which functional associations. The following *Association*-Study seeks to explicitly describe form-functionality relationships, using verbal descriptions and fitting ratings for functionality categories based on the 12 stimuli that were selected in *Pre-Study 2*.

### Method

#### Participants

Thirty-two participants took part in the main *Association-*Study. They were between 21 and 57 years old (*M*_age_ = 32.3 years, *SD* = 3). Thirteen participants were female. All the participants worked in the automotive sector. The participants were naïve to the goals of the study. Thirteen participants had prior experience with haptic technology. There was one left-handed participant in the study. Seven participants took part in one of the previous studies.

#### Apparatus and Stimulus

The apparatus and experimental setup was the same as in Pre-Studies 1 and 2. The touchbox was fitted to house a single form at a time. The participants explored the form with their dominant hand. A special-purpose keyboard (consisting of number keys 1–7 for respective ratings only) to prevent distraction and an audio-microphone to capture verbal recordings were fitted onto the touchbox. A refined stimulus set, consisting of 12 forms based on the results from the *Similarity*-Study, was used (see Figure 6B).

#### Procedure

The participants were introduced to the procedure of the main *Association*-Study. They were asked to imagine encountering the forms in the context of an automotive user interface. The Main Study consisted of three experimental blocks: *Impression, Association*, and *Fitting* (see Figure 1). The blocks were presented in the same order. All 12 stimuli were presented randomly in each of the three blocks, so the participants touched all the forms three times within a single test session. An audio click indicated the start and the end point of the trial and audio recording. The audio was only recorded in the first two blocks. There was no time restriction in either of the blocks. All haptic forms were presented in a randomized order for each block. The first block *Impression* used a think-aloud method. The participants were asked to verbally describe their initial experiences with the form when touching the form for the first time. The participants were instructed to give spontaneous answers. This block was used as a familiarization block. The second block *Association* also used a think-aloud method to gain insight into the relationship between haptic forms and associated functionalities. In this block, the participants were again asked to freely explore the haptic forms and describe functional aspects they associated upon exploration. This also included how they would interact with the forms in an interface, e.g., by pressing or sliding. The third block *Fitting* used a rating procedure to gain insights into the perceived fitting ratings of the participants of the presented forms regarding the three functionality clusters that were being described in Pre-Study 2. After a haptic exploration period, the participants were given three questions: “How fitting is this form to be used as a control element for (1) on/off, (2) adjusting more or less or (3) selection?.” The participants rated fitting of the three functionality categories, respectively, on a scale from one to seven (1 = *not suitable at all*, 7 = *very suitable*). After completing all the experimental blocks, there was a short post-study questionnaire that asked for any difficulties posed by the experimental design. The participants also had the chance to give any further annotations to the study. A test session lasted about 60 min.

### Data Analysis and Results

During the *Association*-Study, two types of data were recorded: qualitative data covering verbal descriptions from the *Impression* and *Association* blocks and quantitative data covering rating data from the *Fitting* block. The verbal descriptions from each trial were transcribed to be used in further analysis. Verbal descriptions were given in German but will be translated to English in this paper. As the *Impression* block was conceptualized as a familiarization phase, we did not include those descriptions in the further analysis.

#### Association Data

For the preparation and analysis of the qualitative data from the *Association* block, we used a similar approach as Muth et al. (2018). They categorized verbal descriptions of artworks based on predefined categories to derive frequency values for the use of specific descriptive categories. Despite having an already predefined set of functionality clusters from Pre-Study 2, we again reviewed all verbal descriptions. Functional categories that were mentioned by most of the participants were on/off, more or less, and selection. The participants reported, in some cases, that a form does not yield any specific functionality association but might be used as search haptic cues as described by Breitschaft et al. (2019a). The participants also explicitly mentioned that there is no specific functionality at all in some cases. An initial list of categories defined by each of the authors separately was discussed and resulted in the following coding scheme: on/off, more or less, selection, search cues, and no functionality.

**Figure 8:**
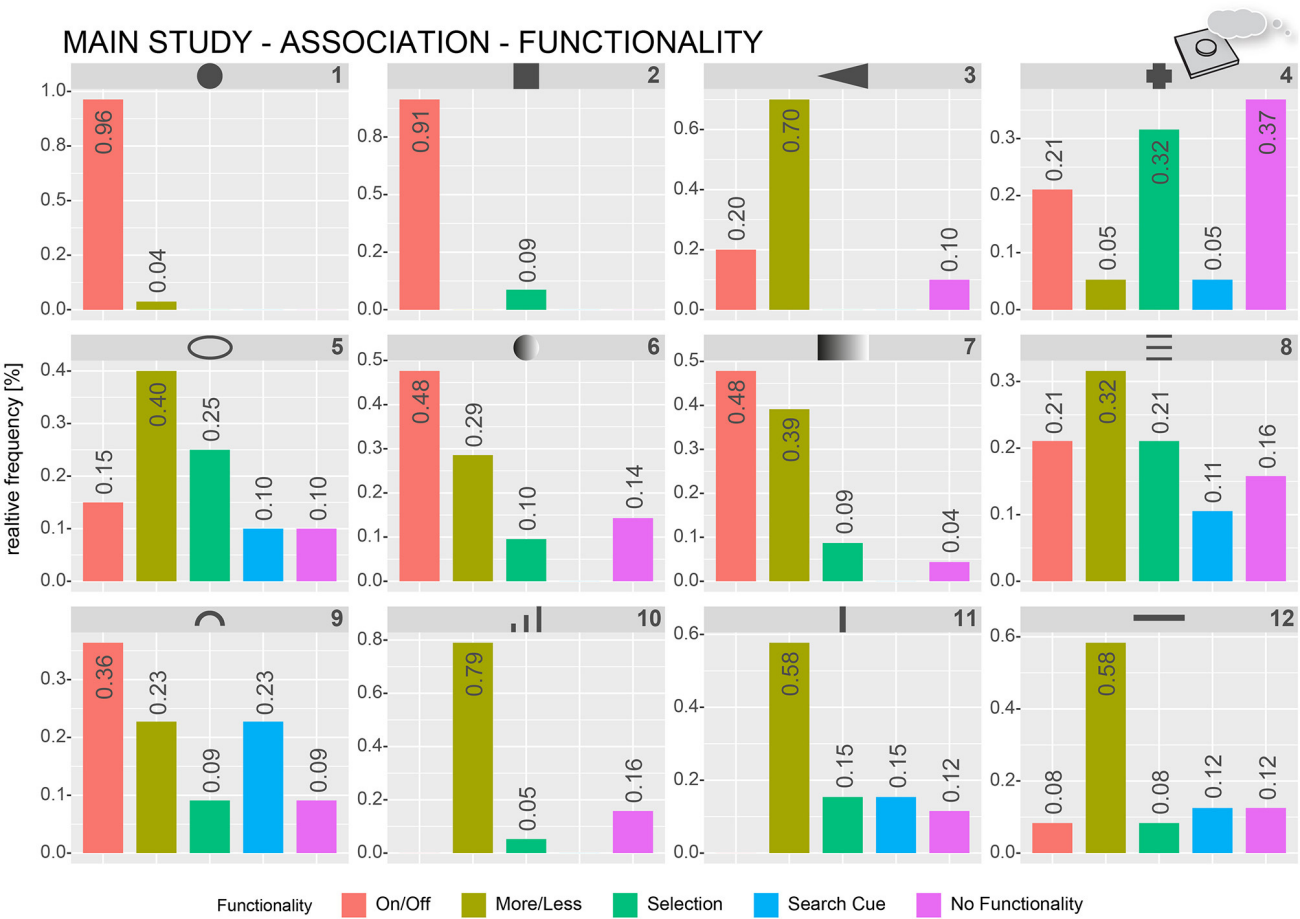
Relative frequencies of how often specific functionality-based categories were assigned by the raters to describe verbal functionality associations of the participants. The numbers represent the relative frequency per category.

Three independent raters categorized each of the verbal descriptions (32 participants × 12 forms = 384 descriptions per rater) based on the created coding scheme. The raters were given an extensive explanation for each of the categories. The raters could freely assign each description to only one category or more than one category. If the participants did not associate a specific functionality, the raters were instructed to assign the “no functionality” category. In total, there were 1,247 category assignments. In 312 cases, all three raters were assigned the same category; in 98 cases, only two of the raters used the same category assignment, and, in 115 cases, only one rater used the category assignments for a specific description. For further analysis, only the verbal descriptions in which all three raters coincided with their category assignment were used. Figure 8 shows the relative frequencies of the functionality-based category assignments per form. For ease of readability in the following sections, form numbers will be given in numbers.

Forms 1, 2, 6, 7, and 9 were most often described by words belonging to the on/off category (e.g., confirmation, button, and push button). Form 1 (circle) and Form 2 (square) were almost exclusively described as being an on/off push button (96 and 91%, respectively). Forms 6, 7, and 9 were mostly being described with respect to on/off functionality (48, 48, and 36%, respectively). The most-used words for describing Forms 3, 5, 8, 10, 11, and 12 were related to more-or-less functionality (e.g., slider, volume, and temperature), ranging from 30 to 79%. The selection-category was the second most assigned functional category for forms 4 (32%), 5 (25%), and 8 (21%). Close to 37% of the descriptions for form 4 included references of the form being useful for detection purposes.

**Figure 9:**
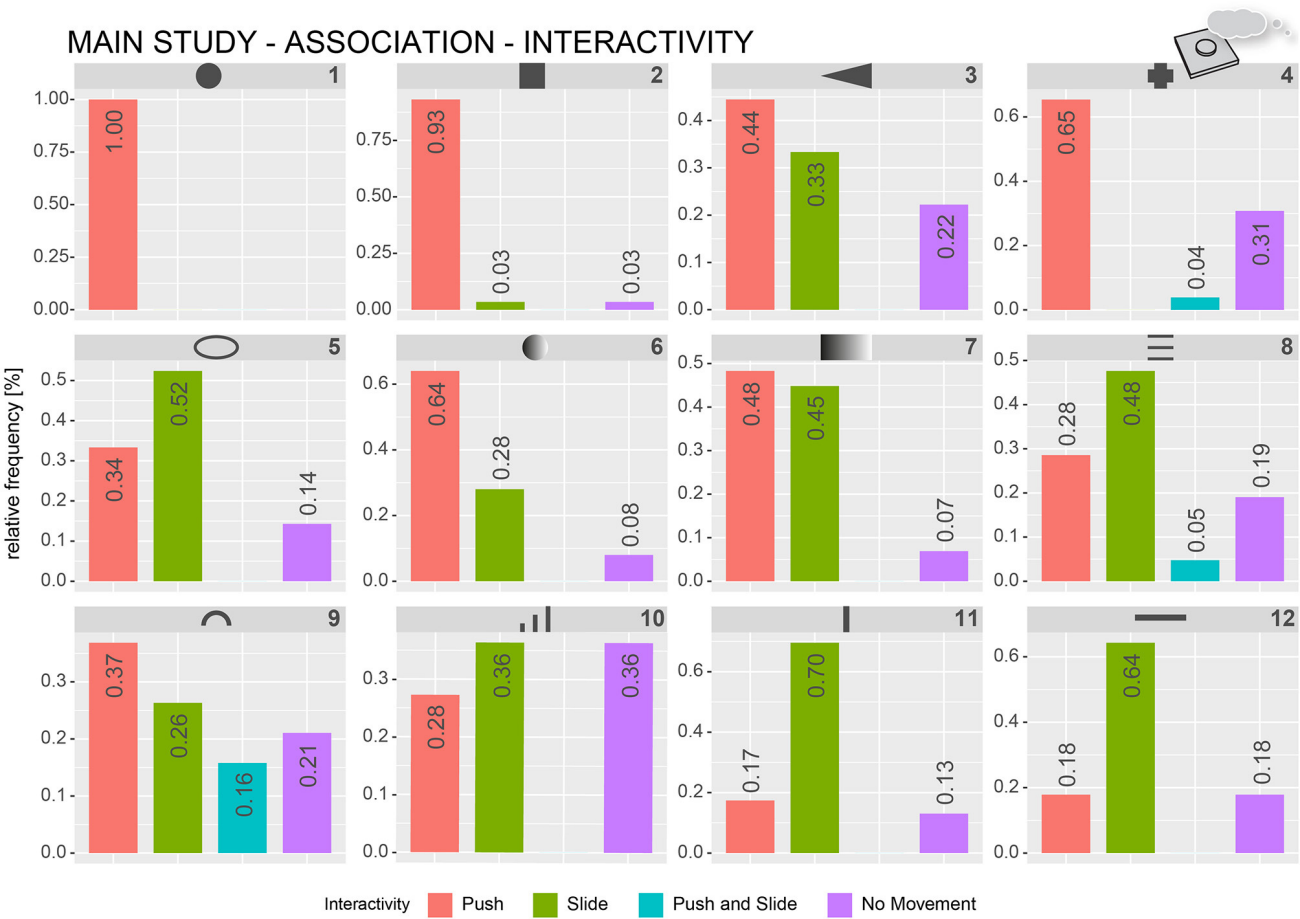
Relative frequencies of how often specific exploration-based categories were assigned by the raters to describe verbal interactivity associations of the participants. The numbers represent the relative frequency per category.

In addition to the perceived functionality, the participants also often described how they would interact (i.e., pressing, sliding, and touching) if the form was represented in a user interface. The previous categorization procedure was repeated with the same three raters and verbal descriptions from the *Association* block to focus on interaction-based descriptions. The coding scheme included the following interaction categories: “push (in z-axis),” “slide (as swiping on a touchscreen),” “push and slide (as moving a physical slider control),” and “no movement.” Figure 9 shows frequency plots of interaction-based category assignments for all forms. Most of the forms, for example, Forms 1, 2, 11, and 12 can clearly be assigned to a single type of interaction. Manipulation by pushing was the most used category for Forms 1 (100%), 2 (93%), 3 (44%), 4 (65%), 6 (64%), 7 (48%), and 9 (37%). Sliding-related descriptions were most used for Forms 5 (52%), 8 (48%), 10 (36%), 11 (70%), and 12 (64%). Form 7 has quite often been described by sliding as well (45%), in addition to pressing (48%). Form 10 was often described as being with no specific movement (36%) as it was with sliding (36%). About 28% would want to use Form 10 by pressing. Form 9 was one of the most ambiguous forms (push, 37%; slide, 26%; push and slide, 16%; and no movement, 21%).

#### Fitting Data

**Figure 10:**
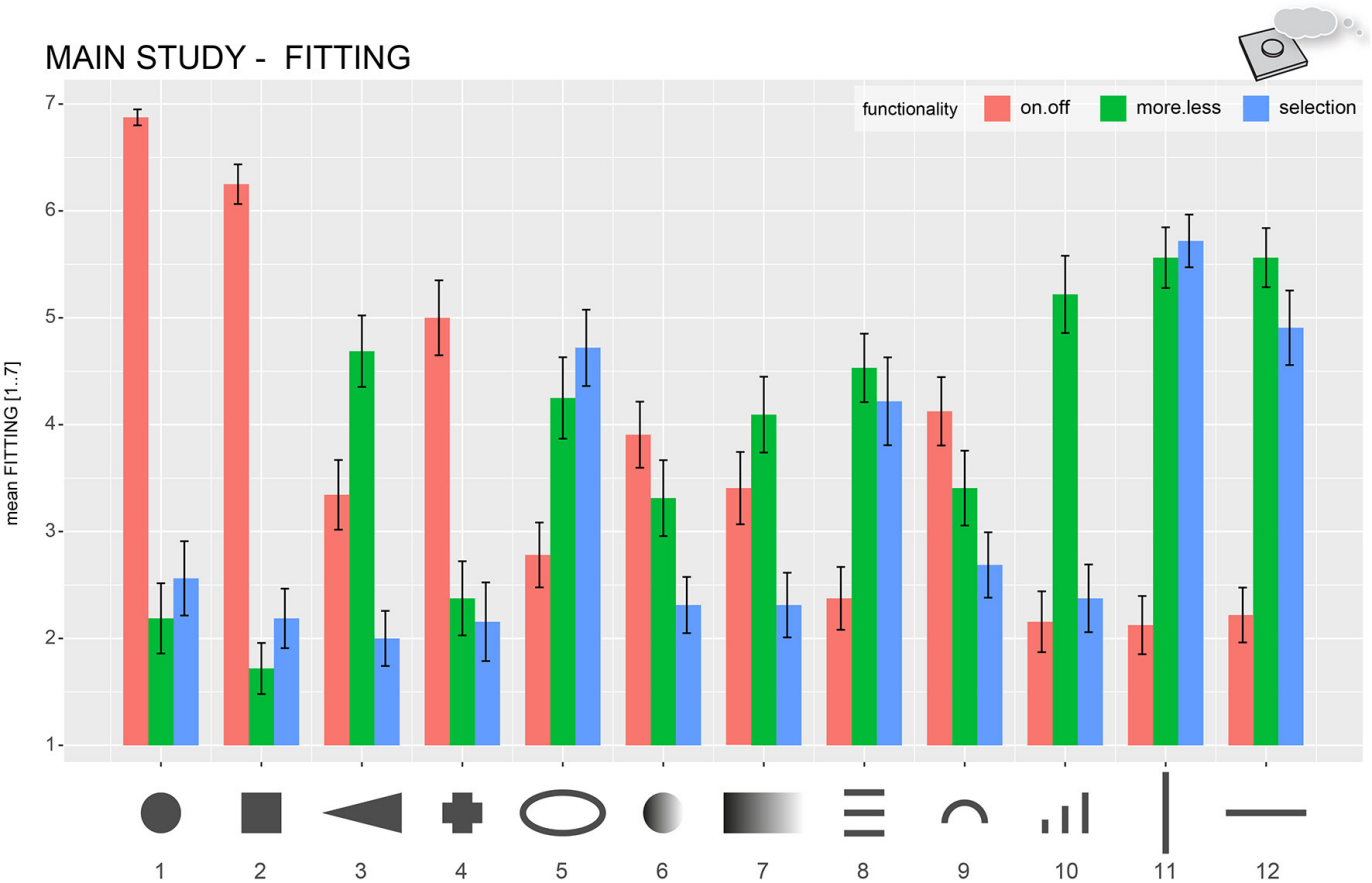
Mean fitting values for all functionality categories (on/off, more/less, selection) for each haptic form. Error bars indicate ± 1 standard error of the mean.

Fitting data from the *Fitting* block were analyzed, using the statistical software R version 4.0.2 (R Core Team., 2021), as well as the *ggplot2* (Wickham, 2016), and *effsize* package (Torchiano, 2020). Figure 10 and **Table 4** show the averaged fitting values grouped by functionality and form. Forms 1 (6.88), 2 (6.25), and 4 (5.00) scored highest on the on/off category, with a high discrepancy to the next functionality category. Form 1 and Form 2 scored the highest mean values overall. Forms 6 (3.91) and 9 (4.12) scored highest on the on/off category but did not seem to differ from the more-or-less category. Forms 3, 7, 8, 10, and 12 scored highest in the more-or-less category (4.69, 4.09, 4.53, 5.22, and 5.56), but only Forms 3 and 10 differed from the next highest category. Forms 5 and 11 scored highest on the selection category (4.72, 5.72), but there were no relevant differences from the more-or-less category.

We established the uniqueness fitting score (UFS) to compare the fitting of specific forms for certain functionalities. It describes the clarity of a form in terms of perceived functionality and is based on the effect size (reported in *Cohen's d*) between the fitting values of the highest and second best-fitting functionality category. The higher the UFS, the clearer association of a form in terms of functionality. The lower the UFS, the more ambiguous the form-functionality association. **Table 4** describes the UFSs as well as the effect sizes to the least fitting category for every form. The UFSs yielded large effects sizes (Cohen's *d* ≥.8), except for Forms 5 (*d* = 0.22, small), 7 (*d* = 0.35, small), 8 (*d* = 0.15, small) 9 (*d* = 0.38, small), 11 (*d* = 0.10, small), and 12 (*d* = 0.37, small).

### Discussion of Form-Functionality Associations

The major aim of the main *Association*-Study was to further explore the general implicit form-functionality pattern found in Pre-Study 2 and describe explicit form-functionality associations. We used a think-aloud method (qualitative) as well as a rating task (quantitative), which were both already used in previous studies, using association-based paradigms (for a more-detailed explanation, see Table 1). In general, combining both data collection strategies yields a strong indication for specific form-functionality relationships. Yet some forms seem to convey clearer functionality associations than others, for example, see Forms 1 and 2 vs. Forms 7 and 9.

Forms 1 and 2 yielded the clearest association pattern. In the *association* as well as the *fitting* task, the protruded square and circle forms yielded the highest scores in the on/off category (see Figures 8, 10) and the highest UFS (each > 3). In most cases, the participants wanted to press these forms (see Figure 9). Typical descriptions included “it just feels like a normal button” or “It's a protruded form I would just like to press.” The protruded circular and squared form seems to strongly resemble a “traditional button.”

Form 3 displayed a more/less functionality for most of the participants (UFS = 0.72). As opposed to most of the other more/less-associated forms, it seems to be ambiguous regarding interaction. Roughly 40% would press, while roughly 33% of the participants would slide to adjust values. The triangular form potentially reminded the participants of a volume element (“It reminds me of increasing/decreasing as the shape converges to a point.”).

Form 4 (cross) seems to be clearly associated with an on/off functionality (UFS = 1.33) that is activated by pressing (65%). However, the verbal associations also pinpoint a selection functionality (32%), as the cross seems to remind the participants of the control pad on a Gameboy. The selection can be made by pressing on one side or the other. Yet the *Perception* Study showed that this form is hard to recognize, which potentially leads to a lot of people having no specific functionality association at all (37%). For the other participants, a mere protrusion may have yielded the association of something that can be pressed—no matter the form.

Form 10, with its triangular geometrical shape, clearly indicated a more/less functionality. The UFS (1.48) indicates a large effect. In almost 80% of the cases, the participants described Form 10, using a more/less functionality. For some participants, the tripartite structure (multiple raised lines increasing in size) yielded an even more detailed association with distinct and increasing levels of intensities (“3 stages,” “*3 different intensities*”; see Table 3). However, interactivity seems to be ambiguous. A third of the participants would like to slide; another third did not report any interaction movement, whereas roughly 25% reported they would want to press.

**Table 3:** Exemplary verbal descriptions by the participants in the *Association* Study.

**Form**		**Exemplary descriptions**
1	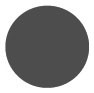	“A protruded area. Just a simple button,” “A circle. I would just like to press it”
2	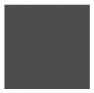	“You'll get stuck on the protruded surface,” “I just want to press it, because it's protruded.”
3	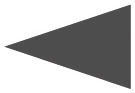	“The triangle is a complicated shape,” “It reminds me of increasing/decreasing as the shape converges to a point,” “could be used for climate control or volume.”
4	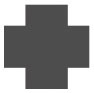	“it' complicated,” “reminds me of a joystick and directional pad,” “could be an iDrive replacement”
5	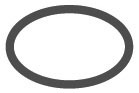	“it's exactly like the click-wheel of the first iPods,” “could be used for navigating in a center display,” “multifunctional,” “contours invite sliding,” “pressing in-between contours”
6	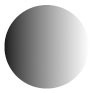	“slide from left to right,” “I would like to slide along the rising area,” “The edge invites pressing,” “it's like sliding and then pressing at the end”
7	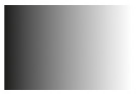	“intensity adjustment,” “reminds me of a rocker key,” “sliding toward rising area,” “I am tempted pressing the protruded edge”
8	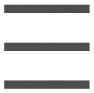	“slide control to open the sun-roof,” “menu-button,” “reminds me of a knurl,” “3-staged element,” “haptic separation between buttons”
9	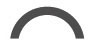	“like a dead-end for the finger,” “finger guidance,” “slide on half-moon,” “natural contour for button area,” “the arc feels like search cue”
10	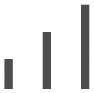	“3 stages,” “more/less,” “WiFi-Button,” “Volume- control,” “three increasing buttons,” “three different intensities”
11	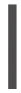	“guidance,” “increase/decrease,” “slider,” “separation between buttons”
12	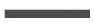	“temperature,” “volume,” “climate control,” “slider,” “guidance and orientation for the finger,” “more/less,” “zoom in/out on a map”

*The number of forms corresponds to the forms in Figure 6B. Gray values correspond to protruded areas*.

Forms 6 and 9 are quite similar with respect to their association patterns. Their UFS ranges between 0.32 and 0.38, which represents a small effect. The on/off category is the most fitting functionality category for Forms 6 and 9. Almost 50% described Form 6, using on/off related, while roughly 30% used more/less-related descriptions. Form 9 seems to be even more ambiguous. About 36% used on/off related, and 23% used more/less-related descriptions. The same number of the participants frequently reported this form in conjunction with being a mere search cue with no further functionality. A similar pattern can be observed for Forms 7 and 8, only that the most fitting category was more/less (UFS = 0.35 and 0.15, respectively). Judging from the qualitative association data, Form 7 was frequently associated with an on/off (48%) as well as a more/less functionality (39%). Interactivity descriptions for Form 7 show an equally ambiguous pattern for pressing (48%) vs. sliding (45%). Although some of the participants reported associating Form 8 with a “burger menu icon” was mostly connected to a more/less and selection functionality that is primarily activated by sliding (48%). The ambiguity of Forms 6, 9, 7, and 8 possibly results from the integration of multiple signifying design features. The participants described the gradient of Form 6 to have the form of a “ramp that you just want to swipe up.” However, the edge of the ramp also invited the participants to push (“I want to make the form flush”). The physically longer gradient in Form 7 seemed to reinforce the sliding and, hence, the more/less impression of the form. Form 9 seems to integrate a mixture of possible signifying design features as well. The half-cut circle clearly represented the boundaries of a button for several participants: “It's like a dead end for the finger,” “finger guidance,” “the arc feels like a search haptic cue.” The “half-moon” created a “natural contour for a button area,” which probably invites users to press. However, in some cases, the arc-like appearance was also associated with a two-way more/less functionality (“slide on half-moon”).

Forms 5, 8, 11, and 12 are among the lowest UFS (0.22, 0.15, 0.10, and 0.37). Each of the forms scored low on the on/off category and high on both the more/less and selection functionality categories with regard to its mean fitting rating. All those forms have in common that almost half of the participants described an interaction *via* sliding during exploration. Forms 11 and 12 were characterized by sliding by 70 and 64% of the participants. Generally speaking, raised-line forms, regardless of their appearance (horizontal, vertical, or a closed circular form), are likely to be interpreted as slider elements. The participants describe protruded contours as “anchors” or “guidance and orientation for the finger.” Looking at the qualitative data in Figure 8, all forms show higher frequency values for the more/less than the selection category. The elliptical shape of Form 5 reminded the participants of the circular click-wheel on older iPods used for scrolling playlists. Although some participants interpreted Form 8 as a “burger menu icon” or a push button with three discrete intensities, most of the participants would use the three horizontally arranged lines as a slider to either scroll lists or adjust temperature and volume. The participants were reminded of a flat and modern-looking interpretation of a knurl element that can regularly be found as a scroll element on automotive steering wheels. The vertical and horizontal arrangements of Forms 11 and 12 both seem to trigger the association of a slider to scroll lists or adjust a more/less continuum. We assume the functionality itself depends on the implementation into the user interface.

## General Discussion

The major aim of this study was to explore functional-based associations of haptic forms based on a highly systematic tripartite (*perception, similarity, association*) multi-study approach. Both Pre-Studies were mainly used to select a set of distinguishable and recognizable forms to be used in the Main Study. Even though having a preliminary character, both Pre-Studies yield insights and prerequisites for examining form-functionality associations. With an open-ended, exploratory approach, we aimed to apply the so-called “aesthetic association principle,” which is one of the basic principles of psychology (1) to a purely haptic domain and (2) in a utility-based rather than an aesthetic-based setting (Carbon and Jakesch, 2013). By doing, so we advocate the integration of a deeper psychological turn in haptic interface design (Carbon, 2019).

### Form-Functionality Associations

**Figure 11:**
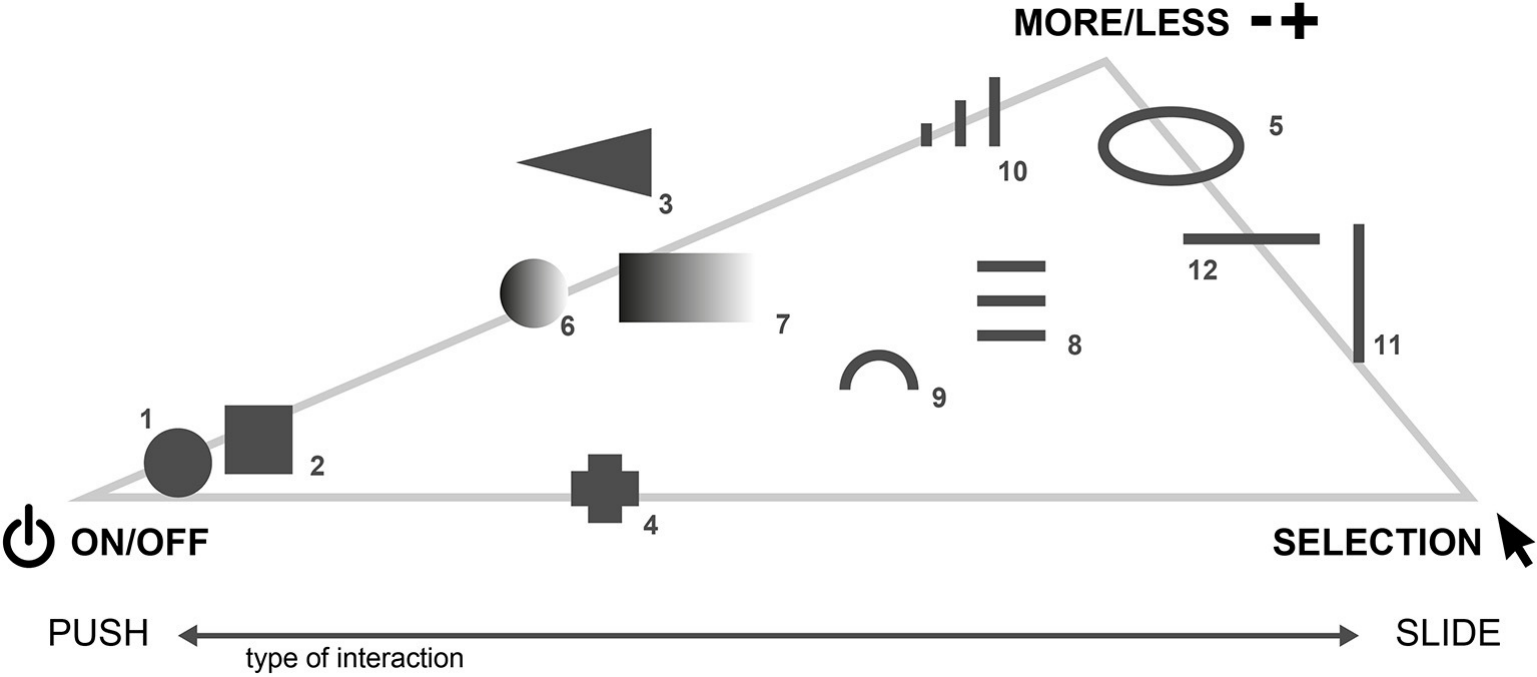
Form-functionality associations based on the forms and functional categories in the Main Study. Some shapes also show a clear tendency toward how the participants would want to interact.

One general conclusion is that haptic forms in a user interface context seem to convey signifying functional aspects (see Figure 11). At a first glance, this seems quite intuitive, but it is important to realize that, first of all, we have to test this intuitive thinking by means of systematic empirical testing. Even more important, this routine has proved to be of great assistance to detect clusters of associations. The MDS procedure in Pre-Study 2 suggests functional-based dissociation of different forms based on four implicit clusters, which we interpreted regarding the following functional categories: on/off, more or less, selection, and unspecified. The verbal association and fitting data from the main *Association*-Study (study 3) revealed explicit form-functionality associations, indicating communicative characteristics of haptic design features. For a detailed description and discussion of association patterns, see section Procedure. In the following section, form numbers refer to the forms in the *Association* Study.

Some forms and haptic design features revealed a strong association with perceived functionality, regardless of interactivity. Forms 3 and 10, both with a triangular shape, were associated mostly with a more/less functionality as it seems to remind the participants of a typical volume symbol. Also, the circular and rectangular shapes (Forms 1 and 2) seem to clearly indicate an on/off functionality. Other forms yielded a strong invitation for interaction but are potentially ambiguous toward functionality. A protrusion—either in the form of fully protruded form (as in Forms 1 and 2) or single salient edges (as in Forms 6 and 7)—seems sufficient to convey a push functionality. Forms including elongated raised lines, such as Forms 11 and 12, clearly afford a sliding interaction no matter the functionality (“It gives guidance and orientation for the finger.”). The Creative Zen Vision: M and the Toshiba Gigabeat MP3-Players are examples of how forms have already been used to facilitate a scroll interaction by sliding (Saffer, 2009).

We suppose the functionality association is not entirely triggered by geometrical shape alone but depends on integration with other design features. Forms 6 and 7 both incorporated a protruded edge and a prolonged “ramp-like” gradient. The edge was often interpreted as a call to push (“I am tempted to make the edge disappear.”), while the gradient invited the participants to slide (“I would like to slide toward the elevation.”), making the form somewhat ambiguous. Possibly, ambiguous forms, such as Forms 6, 7, and 9, incorporate a multitude of signifying design elements, which fail to pronounce a “key interaction.” Indeed, haptic forms that entailed a distinct protruded element, such as Form 1 or “[the] ramp with an edge” (Forms 6 and 7), made the participants frequently think of “something [they] would like to make flush with the surroundings.” Data indicate that the longer “ramp-like” gradient in Form 7 affords sliding as well as more/less impression, while the “edge of the ramp,” which is more pronounced in form 6, invites pressing. The implementation of specific design features, such as size, gradient, and protrusion already seem to pose an interactive context that facilitates or suppresses the functional character of specific geometrical shapes.

This research incorporated simple implementations of the forms, which only provided a generic interaction context (e.g., location of the interface, integration of forms into the interface possible interaction with a GUI, etc.). The forms were presented individually and not embedded in a physical interface (see Figure 2). We suppose that associative strength is influenced by the overall haptic impression of a physical interface—a localized haptic context. Protruded elements seemed to be connected to a push functionality. This is, at least, partly, due to the saliency of the protrusion in an otherwise flat surface. Salient stimuli are said to be highly efficient and informative in perception as they grab attention (Itti, 2007; Kerzel and Schönhammer, 2013), which makes them a value source of information. A “bumpier” interface might decrease the saliency of a form and suppress its associative strength. The same applies to triangular forms (which indicated a more/less functionality). In the presence of a bumpier surface geometry, they blend with the rest of the interface and lose their associative strength. Context variables, such as the implementation of functional elements within non-functional parts of the user interface elements and the interplay of haptic information with multisensory, especially additional visual information like symbols information, impact saliency and thus associative strength. Being aware of modulating factors is essential during the haptic design process to avoid ambiguity and facilitate the intended functional interpretation (Jakesch et al., 2011; Carbon and Jakesch, 2013). Further studies may investigate the modulating influence of additional context information, such as visual symbols on the perceived affordance of haptic forms.

In general, the participants found it difficult to differentiate between a more-or-less and selection functionality (indicated by a small UFS) for some haptic features. The given context and thus associative strength of the forms were probably too weak or too ambiguous to elicit a clear association. Even though Forms 5, 8, 11, and 12 are highly associated with a sliding interaction and more/less functionality, the UFS is low for all of these forms (0.22, 0.15, 0.10, and 0.37). Effect sizes compared with the least fitting the on/off category were high for all those forms (1.03, 1.24, 2.44, and 2.21; see Table 4). The raised-line contours clearly afforded a sliding interaction regardless of the underlying functionality (“finger guidance,” “you snap onto the contour”). Hence, the raised-line forms seem to incorporate user expectation and a stronger underlying interactive metaphor toward interaction rather than functionality. Mlakar and Haller (2020) already proposed to use a straight stitched line to indicate an interaction *via* sliding. The convergence of more/less and the selection functionalities might also be undermined by the conceptual similarities between both functionalities. While the more/less functionality is characterized by the incremental adjustment of ordinally scaled values (e.g., temperature/volume) on a dimension with two extremes (min/max), the selection is characterized by adjusting nominal data in a list. Despite the conceptual overlap, above-mentioned forms are highly suitable in interface contexts, as the context and consequently associative strength toward a specific functionality can be modulated *via* additional graphical information. Forms may probably be interpreted as more/less functions if the form is implemented as a standalone interface element, e.g., like temperature sliders in the VW ID.3 (Volkswagen., 2020) or the molded touch modules to control airflow temperature in the BMW 7 Series (BMW Group., 2019). An additional screen might afford a scrolling/selecting type of interaction, comparable to an automotive rotary controller or the Click Wheel on the early iPods.

**Table 4:** Uniqueness fitting score (UFS) and effect sizes for fitting ratings for each of the forms across the functionality categories on/off, more/less, and selection.

**Form**	**Most fitting**	**Mean**	**Least fitting**	**Mean**	***Effect size***	**2nd best fitting**	**Mean**	***UFS***
1	On/Off	6.88	More/Less	2.19	3.48 *(H)*	Selection	2.56	3.03 *(H)*
2	On/Off	6.25	More/Less	1.72	3.75 *(H)*	Selection	2.19	3.04 *(H)*
3	More/Less	4.69	Selection	2.00	1.60 *(VL)*	On/Off	3.34	0.72 *(M)*
4	On/Off	5.00	Selection	2.16	1.40 *(VL)*	More/Less	2.38	1.33 *(VL)*
5	Selection	4.72	On/Off	2.78	1.03 *(L)*	More/Less	4.25	0.22 *(S)*
6	On/Off	3.91	Selection	2.31	0.98 *(L)*	More/Less	3.31	0.32 *(S)*
7	More/Less	4.09	Selection	2.31	0.96 *(L)*	On/Off	3.41	0.35 *(S)*
8	More/Less	4.53	On/Off	2.38	1.24 *(VL)*	Selection	4.22	0.15
9	On/Off	4.12	Selection	2.69	0.81 *(L)*	More/Less	3.41	0.38 *(S)*
10	More/Less	5.22	On/Off	2.16	1.67 *(VL)*	Selection	2.38	1.48 *(VL)*
11	Selection	5.72	On/Off	2.12	2.44 *(H)*	More/Less	5.56	0.10
12	More/Less	5.56	On/Off	2.22	2.21 *(H)*	Selection	4.91	0.37 *(S)*

*UFS, uniqueness fitting score. Mean values range from 1 to 7. Effect sizes and UFS are reported in Cohen's d. The higher the UFS score, the clearer functionality association of a form. d ≥ 0.2 = S/small, d ≥ 0.5 = M/medium, d ≥ 0.8 = L/large, d ≥ 1.2 = VL/very large, d ≥ 2 = H/huge*.

Interestingly, for some of the forms, the participants already described very specific functional associations. Form 10 reminded some of the participants of the cell reception icons on their phones. It thus represented “setting up a wireless connection.” We argue that some frequently used symbols in graphical interfaces, such as a prototypical antenna symbol or a musical note, could also be used as haptic shortcut elements for functions, such as connectivity or entertainment. Haptic symbols or pictograms have shown to improve the accessibility of interfaces for visually impaired people (Harder and Michel, 2002; Gual et al., 2015).

The required precision for a robust recognition of haptic icons is still subject to discussion. Mlakar and Haller (2020) used familiar UI-symbols (star, home, phone, and heart) that were stitched onto a textile and concluded, that while the star was recognized best, the participants found it rather difficult to correctly name the symbol after eyes-free exploration. Ueda et al. (2016) propose a haptic symbol size of around 120–150 mm^2^ for optimized recognizability. Results from the *Perception*-Study indicate that drawing and thus recognition performance is worse with more complex forms (for an example, see Figure 4), which possibly applies to complex haptic symbols as well. Weak discriminability and recognizability might also decrease the associative character of haptic symbols. We advise designers to use basic and simple forms to ease discriminability and thus maximize saliency in contexts that require efficient and easy-to-use interfaces.

We followed a bottom-up study approach to explore user associations. We examined associative descriptions, using a think-aloud method based on a set of easily recognizable and distinguishable forms whose selection process we described in detail. A more top-down approach has been followed by Van den Bogaert and Geerts (2020) as well as Wobbrock et al. (2009). They used a so-called “end-user elicitation study” to examine affordances of gesture-specific mid-air-haptic feedback and gestures for surface computing. Villarreal-Narvaez et al. (2020) provide an extensive review of gesture elicitation studies. A similar approach was used by Ali et al. (2019) but added an additional evaluation loop and fed back user-elicited symbols in a separate study back to the participants with the task to identify the most fitting representatives for specific certain UI categories. Carbon (2019) emphasized that evaluation of novel and unfamiliar products requires user familiarization to gain valid data on longer-termed user experiences. This might be a limiting factor in elicitation studies: “If you ask people about the future, they will talk about the world of today” (Carbon, 2019, p. 6). Our bottom-up oriented approach seems to be an effective yet convenient and easy-to-execute task for the participants as it focuses on personal learning history of the participants. Experimental data generated by employing the AAP is concrete and allows for direct application. Yet we encourage designers to apply the AAP to their specific field application (visual, as well as audio design) to derive use-case specific guidelines.

### Toward a Deeper Psychological Turn in Haptic Design

Insights from the *perception-, similarity-*, and *association*-study underscore the notion of Carbon notion of the subjective, predictive, dynamic, experience-based, context-sensitive, task-dependent, associative, and multisensory nature of perception (Carbon, 2019). We propose the AAP to be an important and fundamental underlying psychological principle, contributing to the formation of affordances through constant perception, evaluation, and integration of experience. Being aware of the associative relationship between design features and functionality fosters interface design from a functional as well as aesthetical perspective. It also means gaining a deeper understanding of the expectations of users. Creating predictable interfaces by facilitating user associations alleviates cognitive demand and positively influences customer satisfaction (Pohlmeyer et al., 2009). It also complies with one of the basic usability parameters “conformity to expectations” (Heimgärtner, 2017). Future studies might want to explore factors facilitating and suppressing the adoption of associative relationships. In this respect, research should take an even closer look at the role of context (“Are there context-insensitive functional associations?,” “How does additional visual information, such as the presence of a screen, change the functional affordance of a haptic form?,” etc.) and feedback on the adaptation of interaction habits (“Which type of feedback facilitates establishing functional associations?”).

Understanding the experiences of users (i.e., the personal learning history) with haptic interfaces is especially important in understanding the metaphorical value of traditional analog interfaces and how they can be transferred to the digital age. The AAP enables practitioners to gain insight into metaphors governing this switch from analog to digital interfaces, for example, how to design active haptic impulses to retain the rich tactual experience known from traditional button interfaces. Heijboer et al. (2019a) applied the AAP to examine how participants interpret a broad number of piezo-actuated impulses. Also, Breitschaft and Carbon (2020a) aimed to identify haptic analogies in the context of electrostatic friction displays. Form 5 (an ellipse with a raised-line contour) was often associated with the Click-Wheel rotary control found on an early version of the iPod (Apple, 2020). Some participants suggested using such the “elliptical ring” as a modernized interpretation of traditional in-vehicle rotary knobs. Interestingly, something similar happened when the participants explored form 8, which looks like a haptic symbol of a “burger” menu button (which, indeed, was a common association, “that's like a menu shortcut”). Yet the associative data indicate that the participants are more likely drawn toward interaction by sliding. The verbal data reveal that some participants compared form 8 with a reminiscence of a classic “knurl” —comparable to visual signifying cues described in Götz (2007).

The concepts we employed in the study partly remind us of the notions of Gibson and Norman of “affordances.” In a later article, Norman (2008) advocated for using “signifying design features” instead of “(perceived) affordances” to refer to the usability of products to emphasize their context dependency. However, the approach of Norman seems to be more prominent for a posteriori explanation of usable products in the sense of usability heuristics. The approach we propose in the present paper allows to examine and explain the effectiveness of some haptic forms in user interfaces—the approach also explores user expectations to create more compelling interfaces in the first place. Literature still lacks concrete guidelines on how to mindfully incorporate functionality-driven haptic design features in the design process of high-quality and safe-to-use active and passive haptic interfaces (Breitschaft and Carbon, 2020a; Breitschaft et al., 2020). Our work extends findings on signifying surface features in traditional automotive control elements (Götz, 2007) and will hopefully, also, inspire more work on this important topic.

## Limitations

Despite the systematic approach we applied here in terms of a multistep research process, there are limitations of the study. First and foremost, the forms have not yet been integrated and evaluated in a holistic user interface. Hence, important context cues are missing. During the study, no haptic feedback upon confirmation was given—the forms remained static. This was done because we were only interested in examining the initial identifying character of haptic forms as described in the *Identification* phase of the “Framework of Haptic Processing in Automotive User Interfaces” (Breitschaft et al., 2019a).

Also, the given interaction context was too generic compared with diverse real-world interface context demands (e.g., safety in automotive, experience in CE). Context fundamentally influences user associations. We focused on examining general potentially context-independent functional categories rather than interface-specific functions. Another goal was to demonstrate the general effectiveness as well as the ease of use of the AAP in an interface design context. Nonetheless, the participants reported very specific functional associations for some forms (“WiFi,” “First Aid Button”). Future research needs to examine the influence of different context factors on form-functionality associations.

This study used a limited set of generic forms in the *Association* Study, which needs further adaptation for the application in consumer-ready interfaces and potentially lacked representativeness regarding a fully systematic form-functionality approach. We mainly implemented a prototypical and restricted set of forms due to the overwhelming abundance of physical material properties to create haptic forms. We chose to select the initial set of 80 haptic forms (which seemed to be feasible in pilot studies for Pre-Study 1) also based on experience from tangible user interface experts. We opted to take a more application-focused approach with respect to which forms might be applicable in the future interfaces. Another related limitation refers to the number of stimuli we employed in each of the studies. Pre-Study 1 included 80 forms, which were reduced to 20 in Pre-Study 2. The Main Study used 12 forms. These restrictions were based on methodological reasons. Each of the studies was designed to last about 60–90 min, which we found to be the maximum for the untrained participants in haptic studies. We decided on the number of stimuli based on what seemed to be manageable for the participants within this time frame. Indeed, choosing different quantities of stimuli, or making a different stimulus selection might have ended in different results. A larger number of stimuli per stage might influence the overall quality of the results. However, this research was not intended to exhaustively report form-functionality descriptions but to explore common user associations for frequently implemented prototypical forms, rest their examination on a systematic theoretical foundation, and emphasize the importance of a psychology-based perspective of haptic design. A similar issue regarding a manageable sample size and how to optimize the perceptual space have been described by MacLean and Hayward (2008). We followed the approach of MacLean (2008) and implemented a tripartite perception-based form selection process, which underscores important facets during the haptic design process—*recognition and detectability, distinctiveness*, and *semantic significance*. Further studies might examine functionalities, using broader and more systematically generated stimuli. Pre-Study 1 and Pre-Study 2 included 10 and 14 participants, respectively. The sample size might appear lower than comparable studies but needs to be considered within the context of the entire multistep research approach. The first two studies were designed as Pre-Studies and not as standalone experiments. We described the Pre-Studies in more depth to provide practitioners with an easy-to-implement procedure to support their design workflow. Pre-Study 1, which was based on a qualitative judgment of drawings, might have benefited from a greater number of participants to derive valid design guidelines. Also, the MDS in Pre-Study 2 might have yielded a more precise representation of actual associative patterns. Even though we explore haptic design guidelines, the main goal of the Pre-Studies was to provide a very general foundation for stimulus selection. MacLean and Hayward (2008) describe a similar approach of “perceptually optimizing” a larger set of stimuli within their research on haptic icons. They argue that a few participants might already generate a sufficient amount of dissimilarity data.

## Ideas for Application

The current set of stimuli consisted of a homogenous material in terms of hardness and surface texture. Mlakar and Haller (2020) already examined signifying features in textile interfaces. They showed that surface features, such as stitching, and also concave or convex surface geometries, are associated with a call for action. Götz (2007) gives an extensive overview of visual-based affordances of traditional automotive control panels. Additionally, other haptic material qualities, such as temperature, hardness, and slipperiness, seem promising to be used in UI contexts but have not yet been examined with respect to their signifying character. For an extended review of haptic material qualities, see Bergmann Tiest (2010) and Klatzky et al. (2013). Iosifyan et al. (2017) already found semantic associations between haptic material, such as silk or wood and multisensory stimuli, such as movie snippets. Especially surface features, such as compliance, may elicit a profound “call for pressing” in the z-direction due to the compliant surface material. Besides haptic features, also the dynamics of shape-changing interfaces may be useful for interface design to display system state and an affordance (Tiab and Hornbæk, 2016; Petersen et al., 2020).

Applying a more profound psychological perspective by employing semantic analogies has the potential to benefit a broad spectrum of applications, such as novel haptic technologies and automotive tangible user interface design. Haptic experience constitutes more than a mere technical perspective of haptic technologies. It is embedded into a reciprocal interaction of context, user, and technology. Methodological approaches, such as shown in the *perception-, similarity*-, and *association*-study, promote understanding of the perception of the users of innovative haptic devices (Breitschaft and Carbon, 2020b) and support a truly human-centered approach to haptic interface design (Breitschaft et al., 2020). Automotive user interfaces may especially profit from a cognitive-driven design process as they need to meet a multitude of different requirements, ranging from a safe and efficient interaction to a highly aesthetic appearance. Integrating an association-driven haptic form language into seamless tangible user interfaces may not only enable orientation but also already identification of interface elements, reduce driver distraction by alleviating cognitive demand, and positively impact user experience due to conformity with user expectations (Carbon and Jakesch, 2013; Breitschaft et al., 2019a). It may also guide familiarization and ease of use with novel technologies as association potentially triggers deeply rooted routines (Breitschaft et al., 2019a), especially in cases of eyes-free operation. Future research should aim to test the usability of an association-based user interface in an applied automotive setting. Following an association-based perspective may also improve usability and product appreciation in other domains of haptic design, such as consumer electronics, transportation, virtual reality, and robotics. Even though we focused on form-functionality associations, the *aesthetic association principle* might also be applied in haptic aesthetics to provide a cognitive-driven explanation why certain material properties are appealing to touch.

## Conclusion

This series of studies reinforces the need for the implementation of a stronger psychological and contextual perspective in haptic interface design. We depicted how the mindful integration of psychological paradigms from the early ages of psychology in the nineteenth century, such as *aesthetic association principle*, has fundamental repercussions on new-age design disciplines, like haptic interaction design. Focusing on a psychological rather than purely technical reality opens new perspectives for refining the current design and pathing the way for rethinking and readjusting established design and engineering practices to govern the transformation from analog tangibility to a digital virtuality. The adaptation of psychological paradigms is a key challenge in the human-centered design process. It is an important stepping stone toward successfully following of a holistic “Psychology-of-Design” approach to design.

## Data Availability Statement

The raw data supporting the conclusions of this article will be made available by the authors, without undue reservation.

## Ethics Statement

Ethical review and approval was not required for the study on human participants in accordance with the local legislation and institutional requirements. The patients/participants provided their written informed consent to participate in this study.

## Author Contributions

SB and C-CC had the initial idea to apply the aesthetic association principle to the haptic interaction domain and worked further on the manuscript. SB conceptualized the design of experiments, manufactured haptic stimuli, did the main part of the research, conducted the studies, mainly wrote the manuscript, and created graphical content. Both authors contributed to the article and approved the submitted version.

## Conflict of Interest

At the time of researching and writing this paper, SB was enrolled in the PhD program at BMW Group and Bamberg Graduate School of Affective and Cognitive Sciences (BaGrACS), supervised by C-CC.
